# Untargeted Metabolomic Profiling of Spermatozoa and Seminal Plasma Identifies Candidate Metabolic Indicators of Semen Quality in Holstein Bulls

**DOI:** 10.3390/ani16193098

**Published:** 2026-10-02

**Authors:** Olga Kostyunina, Alexey Traspov, Anton Ermilov, Faridun Bakoev, Gleb Nemkovskiy, Alexander Kusnetzov, Vladimir Belyakov

**Affiliations:** LLC «WESTTRADE Ltd.», 115201 Moscow, Russia; traspovalex@gmail.com (A.T.); de_lamak@mail.ru (A.E.); bakoevfaridun@yandex.ru (F.B.); gbnem@mail.ru (G.N.); kuz.ab@icloud.com (A.K.); bel.vk@yandex.ru (V.B.)

**Keywords:** Holstein bulls, semen quality, spermatozoa, seminal plasma, metabolomics, LC–MS/MS, metabolomic profiling, amino acid metabolism

## Abstract

This exploratory study applied untargeted LC–MS/MS metabolomic profiling to investigate metabolic differences between Holstein bulls with high-quality (HQ) and low-quality (LQ) semen in both spermatozoa and seminal plasma. Bulls were classified based on long-term routine semen quality records and ejaculate rejection rates, providing a stable and biologically meaningful grouping. A total of 249 unique metabolites were annotated across both biological matrices. Differential analysis identified 46 LC–MS features corresponding to 44 unique metabolites in spermatozoa and 11 LC–MS features corresponding to 9 unique metabolites in seminal plasma that met the predefined multi-criteria selection thresholds (FDR < 0.05, |log_2_FC| > 1, VIP > 1, and AUC ≥ 0.80 or ≤0.20). The HQ group was characterized by higher levels of metabolites involved in amino acid and energy metabolism, including glutamine, glutamate, aspartate, succinate, ornithine, citrulline, and arginine. Conversely, the LQ group showed increased abundance of metabolites potentially related to altered redox homeostasis and compounds potentially originating from dietary or microbial sources. As this is an exploratory study, mechanistic interpretations regarding oxidative stress, microbiome-derived metabolites, and actual fertility should be considered strictly hypothesis-generating. The annotated metabolites represent candidate indicators associated with semen quality parameters rather than validated biomarkers. Their relationship with actual field fertility and their underlying biological mechanisms require confirmation in larger, independent populations with direct functional assays and field fertility records.

## Simple Summary

Reproductive efficiency is crucial for the economic success of dairy farming. Because a single bull can inseminate thousands of cows, poor semen quality leads to significant financial losses. However, standard evaluation methods cannot fully explain why some bulls produce better quality semen than others. This study aimed to uncover the underlying biological differences by examining the chemical composition of both sperm cells and the surrounding seminal fluid from Holstein bulls. We used advanced chemical analysis to measure small molecules, known as metabolites, in samples from thirty bulls classified as having either high-quality or low-quality semen based on long-term records. As a result, we identified 46 LC–MS features (44 unique) in spermatozoa and 11 features (9 unique) in seminal plasma. Bulls with high-quality semen consistently showed higher levels of molecules involved in cellular energy production and protein building blocks. These findings suggest that specific chemical signatures are strongly linked to better semen quality. Ultimately, this research provides new insights into reproductive biology and could eventually help breeding programs select superior bulls, improving the sustainability and profitability of the global dairy industry.

## 1. Introduction

Reproductive efficiency is a key determinant of the economic sustainability of dairy cattle production. Artificial insemination is the predominant reproductive technology in modern cattle breeding, enabling the widespread use of semen from genetically superior bulls [1]. Because a single bull contributes to the insemination of numerous cows, reduced semen quality may adversely affect breeding efficiency and increase economic losses associated with discarded ejaculates and replacement of breeding sires [2,3]. However, conventional semen evaluation methods provide limited insight into the molecular mechanisms underlying these differences in semen quality, and the metabolomic basis of this variation in Holstein bulls remains incompletely understood. Furthermore, in the context of commercial artificial insemination, stringent semen quality standards are economically and biologically critical. High-quality ejaculates can be extended into hundreds of doses, maximizing genetic impact, whereas suboptimal semen often exhibits poor cryotolerance and higher rates of DNA fragmentation, leading to early embryonic loss and reduced conception rates.

Semen quality is a multifactorial trait influenced by both genetic [4] and non-genetic factors, including nutrition, age, management, and housing conditions [5]. Routine semen evaluation is primarily based on sperm concentration, motility, morphology, and other standard quality parameters. Although these assessments are essential for semen quality control, they may not fully explain the biological basis of variation among bulls [6]. Metabolomics has emerged as a promising approach to characterize the biochemical composition of spermatozoa and seminal plasma, offering the potential to identify metabolic signatures associated with semen quality and overall reproductive performance.

Spermatozoa and seminal plasma contain numerous biologically active molecules that contribute to sperm function. Seminal plasma proteins and metabolites participate in the regulation of sperm motility, capacitation, protection against oxidative damage, acrosome reaction, and other physiological processes involved in successful fertilization [7,8]. Consequently, alterations in the metabolomic composition of spermatozoa and seminal plasma may reflect differences in semen quality and provide insights into the metabolic processes underlying sperm function. In addition to metabolomic approaches, transcriptomic [9] and proteomic [10] studies have contributed to our understanding of the molecular basis of sperm function and male fertility.

Among omics technologies, metabolomics offers a comprehensive approach for characterizing low-molecular-weight metabolites that represent the downstream products of cellular metabolism [11,12]. Because metabolites directly reflect the physiological state of cells and tissues, metabolomic profiling has become an important tool for investigating biological processes under both normal and altered physiological conditions [13]. In reproductive biology, metabolomics has increasingly been applied to characterize the biochemical composition of spermatozoa and seminal plasma and to identify metabolic pathways associated with sperm physiology. For example, studies in mouse models have demonstrated the importance of glycolytic and mitochondrial pathways in sperm function [14].

Several studies have investigated the metabolome of bovine spermatozoa and seminal plasma using gas chromatography [15,16] and liquid chromatography coupled with mass spectrometry (LC–MS/MS) [17,18,19]. These studies have identified metabolites associated with sperm motility, morphology, and other semen quality characteristics. However, the reported metabolite profiles remain heterogeneous across studies because of differences in analytical platforms, sample preparation, and experimental design. Importantly, most previous studies have focused on either spermatozoa or seminal plasma in isolation, and relatively few have performed simultaneous untargeted profiling of both biological matrices from the same bulls. Moreover, to our knowledge, no previous study has comprehensively compared the metabolomic profiles of both spermatozoa and seminal plasma in Holstein bulls classified according to long-term routine semen quality records and ejaculate rejection rates, which provides a more stable and biologically meaningful classification than single-ejaculate assessments.

To address this knowledge gap, the aim of the present study was to perform comparative untargeted metabolomic profiling of spermatozoa and seminal plasma from Holstein bulls with high-quality (HQ) and low-quality (LQ) semen, using long-term production records to define semen quality groups. To our knowledge, this is the first study to integrate paired untargeted metabolomic profiling of both spermatozoa and seminal plasma with long-term semen quality classification in Holstein bulls, providing a comprehensive view of the metabolic pathways associated with semen quality. By integrating metabolomic information from both biological matrices, this study sought to identify metabolic signatures and candidate metabolic indicators associated with semen quality, thereby providing a more comprehensive understanding of the metabolic pathways underlying variation in this important economic trait in Holstein bulls.

## 2. Materials and Methods

### 2.1. Animal Management and Semen Collection

The study was conducted on Holstein bulls (*n* = 30) belonging to JSC “Moskovskoe” Breeding Company (Noginsk, Moscow Region, Russia). Semen samples were obtained from breeding bulls during routine ejaculate collection. Since all samples originated from standard production procedures without additional manipulations of animals, ethical approval was not required. All bulls were maintained under identical housing, feeding, and management conditions and received the same standardized diet. Furthermore, to minimize seasonal variations, a single representative semen sample was collected from each of the 30 bulls within a narrow, one-month period during autumn for metabolomic analysis. There were no statistically significant differences in age between the HQ (3.1 ± 1.6 years) and LQ (3.5 ± 1.6 years) groups (*p* = 0.52, as detailed in Supplementary Table S1). While collection followed standard operating procedures, detailed records of the abstinence interval were not available in the retrospective dataset.

### 2.2. Semen Quality Evaluation and Grouping

Based on long-term production records collected during 2023–2024, bulls were assigned to two groups according to semen quality. As part of routine semen evaluation, the breeding center assigns a simplified categorical activity score (0–8) to each ejaculate, with higher scores indicating better overall semen quality. The semen-quality classification used in the breeding program was based on progressive sperm motility and sperm concentration, with progressive motility ≥ 70% and sperm concentration ≥ 0.7 × 10^9^ cells/mL considered indicative of the target semen-quality level. These thresholds are based on the internal Standard Operating Procedures (SOP) of the breeding company, which align with general international industry guidelines for artificial insemination centers to ensure sufficient post-thaw survival and viable sperm count per commercial straw. However, the categorical activity score was not mathematically converted to progressive motility percentages and was not used as a quantitative surrogate for the ≥70% threshold.

Bulls with consistently high activity scores and less than 10% rejected ejaculates over the recording period were assigned to the high-quality group (HQ, *n* = 15), whereas bulls with consistently lower activity scores and more than 30% rejected ejaculates were assigned to the low-quality group (LQ, *n* = 15). Group assignment was based on the long-term production records rather than on the single ejaculate collected for metabolomic analysis, thereby reducing the influence of single-ejaculate variability. At the group level, the HQ bulls exhibited a significantly higher mean activity score (8.0 ± 0.1 vs. 5.8 ± 1.8, *p* < 0.001) and a much lower ejaculate rejection rate (4.4 ± 1.8% vs. 50.4 ± 20.1%, *p* < 0.001) compared to the LQ group. Additionally, sperm concentration was significantly higher in the HQ group (1.66 ± 0.15 vs. 1.49 ± 0.25 ×10^9^ cells/mL, *p* = 0.04), while ejaculate volume did not differ significantly between the groups (5.7 ± 1.2 vs. 6.7 ± 2.6 mL, *p* = 0.14). Based on long-term production records from 2023–2024, a total of approximately 3700 ejaculates were evaluated for the 30 bulls, averaging 133.0 ± 52.0 ejaculates per bull in the HQ group and 113.9 ± 33.8 in the LQ group.

### 2.3. Sample Preparation and Metabolite Extraction

Following semen collection, spermatozoa were separated from seminal plasma by centrifugation at 700× *g* for 10 min at 4 °C. The supernatant (seminal plasma) was carefully transferred to fresh tubes and centrifuged again at 10,000× *g* for 60 min at 4 °C to remove residual cellular material [16]. The sperm pellets were not washed prior to metabolite extraction in order to minimize metabolite loss and preserve the endogenous metabolomic profile during sample preparation, although this approach may have resulted in minor residual seminal plasma contamination [20]. Untargeted metabolomic profiling was performed at the Institute for Molecular Theranostics, Sechenov University. Prior to sample preparation, frozen samples were stored at −80 °C and thawed on ice for approximately 4 h. Metabolite extraction was performed using a fixed sample volume (100 μL) without prior normalization to sperm cell number, protein content, or DNA content. Both seminal plasma and spermatozoa samples were extracted using the same extraction protocol. Each 100 μL sample was mixed with 900 μL of chilled methanol:acetonitrile:water (2:2:1, *v*/*v*/*v*) containing amitriptyline and verapamil (500 ng/mL each) as internal standards. These internal standards were added prior to extraction to monitor sample preparation and instrument performance. They were not used for normalization of metabolite abundances. Instead, signal normalization across samples was performed using Probabilistic Quotient Normalization (PQN) during data preprocessing. Samples were vortexed for 10 s, subjected to five cycles of sonication for 5 s each with cooling on ice between cycles, and then additionally sonicated for 20 min under cooled conditions. Subsequently, samples were incubated on an orbital shaker for 10 min at 5 °C and centrifuged at 13,000 rpm for 10 min at 10 °C. A 600 μL aliquot of the supernatant was transferred to a fresh tube, evaporated under vacuum, and reconstituted in 100 μL of mobile phase B (acetonitrile:water 9:1, *v*/*v*, containing 10 mM ammonium acetate). After reconstitution, samples were centrifuged again for 10 min at 10 °C, and 50 μL of the final supernatant was transferred into chromatographic vials for LC–MS analysis.

### 2.4. Untargeted LC–MS/MS Analysis

Chromatographic separation was performed using an Agilent 1290 Infinity II UHPLC system (Agilent Technologies, Santa Clara, CA, USA) coupled to a Sciex TripleTOF 6600 time-of-flight mass spectrometer (SCIEX, Framingham, MA, USA). Metabolites were separated by hydrophilic interaction liquid chromatography (HILIC) on a SeQuant^®^ ZIC-HILIC column (100 × 2.1 mm; Merck, Darmstadt, Germany). Mobile phase A consisted of water containing 10 mM ammonium acetate, whereas mobile phase B consisted of acetonitrile:water (9:1, *v*/*v*) containing 10 mM ammonium acetate. The injection volume was 2 μL, the flow rate was 0.25 mL/min, the column temperature was maintained at 45 °C, and the autosampler temperature was set to 4 °C. The chromatographic gradient was as follows: 0 min, 99% B; 15 min, 70% B; 15.1 min, 99% B; and 20 min, 99% B, resulting in a total run time of 20 min. Mass spectrometric data were acquired in both positive and negative electrospray ionization modes using information-dependent acquisition (IDA) over an *m*/*z* range of 100–650. The ion source parameters were as follows: IonSpray Voltage +5500 V (positive mode) and −4500 V (negative mode); Curtain Gas 35 psi; GS1 55 psi; GS2 55 psi; and source temperature 550 °C. The collision energy was set to ±35 V (with the sign depending on the ionization mode), and the declustering potential (DP) was ±80 V. Calibration was performed automatically in the Analyst software (v. 1.7.3, SCIEX, Framingham, MA, USA) using external standards (PPG POS solution for positive mode and ESI Negative Calibration Solution for negative mode) between analytical runs and whenever a noticeable mass drift was observed. Instrument mass accuracy was routinely monitored throughout the analytical sequence, and recalibration was performed whenever the manufacturer’s recommended mass accuracy specification was exceeded. Approximately 25% of the samples were analyzed in technical replicates to assess analytical reproducibility.

Raw LC–MS data were processed and metabolites were annotated using MS-DIAL software v. 4 [21], while quantitative peak intensities were extracted using Skyline software (v. 24.1, MacCoss Lab, University of Washington, Seattle, WA, USA) [22]. Metabolite identification criteria included signal intensity > 2000 cps, MS/MS spectral similarity > 0.8, signal-to-noise ratio > 10, and at least a 10-fold higher response in biological samples compared with blank injections. Metabolite annotation was performed using accurate mass, retention time, adduct assignment, isotope pattern, and MS/MS spectral matching implemented in MS-DIAL. Annotation confidence was classified according to the Metabolomics Standards Initiative (MSI) guidelines. Since authentic reference standards were not analyzed, metabolite annotations reported in this study represent putatively annotated compounds (MSI level 2). Mass error was calculated from the difference between the observed and reference *m*/*z* values and is reported for each annotated metabolite in Supplementary Table S2. Annotations with mass errors exceeding ±10 ppm were excluded from the final dataset, consistent with published untargeted LC–HRMS metabolomics workflows [23]. Detailed annotation information, including retention time, observed *m*/*z*, adduct type, molecular formula, mass error, spectral library match, and annotation level, is provided in Supplementary Table S2.

The analytical workflow was organized into four separate analytical batches corresponding to the combination of biological matrix and ionization mode. Within each batch, samples from both HQ and LQ groups were randomized in the injection order to minimize batch effects and analytical bias. Samples were randomized within each analytical batch to ensure balanced representation of HQ and LQ groups across the injection sequence. Pooled QC samples were injected every 10–12 injections to monitor signal stability and analytical reproducibility throughout each batch. System suitability test (SST) samples were included at the beginning of positive ionization mode batches to verify instrument performance prior to sample analysis. Blank samples (extraction solvent without biological material) were processed alongside the study samples and injected periodically in each batch to identify and exclude background contaminants and analytical artifacts.

Prior to statistical analysis, a systematic multi-step filtering workflow was applied to ensure data quality and biological relevance. First, a two-tier classification system was used to distinguish between analytical artifacts/xenobiotics and naturally occurring dietary- or microbiome-derived metabolites. Compounds classified as analytical artifacts or xenobiotics were manually identified based on spectral library matching and manual curation, and were systematically excluded from the dataset. This category included pharmaceuticals and veterinary drugs (e.g., ciprofloxacin, enrofloxacin, lincomycin, meloxicam, propranolol), synthetic pesticides and herbicides (e.g., atrazine, piperonyl butoxide), industrial chemicals and plasticizers (e.g., benzalkonium chloride, phthalates, bisphenol A, triclosan, crystal violet, melamine), detergents and surfactants (e.g., lauramidopropyl betaine, myristamidopropyl betaine, dodecylbenzenesulfonic acid), and laboratory reagents and contaminants (e.g., tributylamine, 1,3-diphenylguanidine, 1,2,3-benzotriazole, 2-naphthylamine, cis-1,2,3,6-tetrahydrophthalimide). These compounds do not naturally occur in biological matrices and were considered analytical artifacts or contamination. In contrast, naturally occurring dietary- and microbiome-derived metabolites (e.g., plant polyphenols such as enterolactone, daidzein; tryptophan-derived indole metabolites such as indole-3-carboxaldehyde and indoleacetic acid; and specific organic acid derivatives) were retained in the final dataset. These compounds represent genuine components of the semen metabolome that reflect the physiological interaction between diet, rumen microbial metabolism, and host metabolism, rather than analytical contamination.

Following this manual curation, additional automated data-filtering steps were applied. First, features with >30% missing values—defined as zero-intensity (non-detected) signals across the 30 biological samples—were excluded. Second, duplicate features, including potential peak-picking or deconvolution duplicates with the same putative annotation and nearly identical retention times, as well as multiple adduct forms of the same annotation, were consolidated by retaining the variant with the lowest missing rate and the highest mean intensity; removed variants are indicated in Supplementary Table S2 under “Reason for exclusion”. Conversely, features with the same putative annotation but clearly different retention times were retained as separate LC–MS features and were not interpreted as the same structurally resolved metabolite in the absence of authentic reference standards. Third, a prevalence filter was applied, requiring each feature to be detected in at least 50% of the samples within at least one experimental group (HQ or LQ). Finally, the remaining curated data were normalized using probabilistic quotient normalization (PQN), followed by log_2_ transformation and Pareto scaling prior to statistical analysis.

Untargeted metabolomic profiling was performed on a cohort of bull semen samples, from which 30 bulls were retrospectively selected for the present study according to predefined semen quality criteria. To prevent analytical bias, all samples were submitted to the laboratory in a blinded (anonymized) manner using coded identifiers. The analytical workflow was organized into batches of approximately 50 injections each; therefore, samples from the HQ and LQ groups were systematically distributed throughout the analytical batches rather than analyzed as separate consecutive groups. Each analytical series began with the injection of five solvent blank samples to monitor background contamination and carryover effects. Additionally, two procedural internal standards (amitriptyline and verapamil) were added to all samples prior to extraction to monitor extraction efficiency and injection reproducibility. Importantly, the metabolomic data were not normalized to these internal standards, as they cannot adequately represent the diverse chemical classes detected in untargeted metabolomics.

### 2.5. Statistical and Bioinformatics Analysis

Statistical analyses were performed in R version 4.3 (R Foundation for Statistical Computing, Vienna, Austria). Prior to multivariate analysis, missing values were imputed using low-abundance estimates (1st percentile), followed by Probabilistic Quotient Normalization (PQN) [24], log_2_ transformation, and Pareto scaling [25]. Prior to supervised modeling, unsupervised principal component analysis (PCA) was performed on the Pareto-scaled data to assess natural clustering, data quality, and the presence of outliers. PCA score plots were inspected to verify that the observed group separation was not driven by analytical artifacts, batch effects, or extreme outliers. To assess group separation and identify metabolites contributing to discrimination between HQ and LQ bulls, partial least squares discriminant analysis (PLS-DA) was performed using the mixOmics package [26]. To mitigate the risk of overfitting, the optimal number of latent components was selected using five-fold cross-validation based on the minimum Balanced Error Rate (BER), which serves as a robust predictive metric for small datasets. Model significance was rigorously evaluated by permutation testing (200 permutations). To prevent circular biomarker selection, Variable Importance in Projection (VIP) scores were not used in isolation. Instead, VIP stability was assessed using 100 bootstrap resampling iterations; metabolites were considered stable candidates only if their VIP score exceeded 1.0 in at least 70% of the bootstrap iterations. Differential metabolite analysis was performed independently using the two-sided Mann–Whitney U test [27], with correction for multiple comparisons conducted using the Benjamini–Hochberg false discovery rate (FDR) procedure [28]. Candidate metabolites were prioritized using a multi-criteria approach, requiring simultaneous fulfillment of the following thresholds: FDR < 0.05, |log_2_FC| > 1, VIP > 1, and VIP stability > 70%. To evaluate the discriminatory power of individual metabolites, the area under the receiver operating characteristic curve (AUC) was calculated. For this analysis, the high-quality (HQ) group was explicitly defined as the positive class (case), and the low-quality (LQ) group as the negative class (control). To account for the direction of association, metabolites were considered to have strong apparent discriminatory power if they showed high predictive value for either group: an AUC ≥ 0.80 (indicating the metabolite is elevated in and predicts the HQ group) or an AUC ≤ 0.20 (indicating the metabolite is elevated in and predicts the LQ group, equivalent to an inverted AUC ≥ 0.80 for the LQ group). To assess the precision of these apparent AUC estimates, 95% confidence intervals (CIs) were calculated for all metabolites using 2000 bootstrap iterations. Metabolites detected in both positive and negative ionization modes were retained as separate entries for differential analysis to preserve the statistical power of each ionization mode. For KEGG pathway enrichment analysis, duplicate entries were collapsed into single unique compound names to prevent artificial inflation of pathway hit counts. For these metabolites, the previously calculated AUC values and their 95% confidence intervals are reported in Table 1 (spermatozoa). The significantly enriched KEGG metabolic pathways for the differential metabolites in spermatozoa are presented in Table 2. The differential metabolites and their statistical parameters in seminal plasma are reported in Table 3.

Biological interpretation of metabolomic alterations was performed using pathway enrichment analysis through the MetaboAnalyst 6.0 platform. The analysis was conducted using the Over-Representation Analysis (ORA) module based on the hypergeometric test, utilizing the reference Bos taurus (cattle) pathway library from the Kyoto Encyclopedia of Genes and Genomes (KEGG) database. The input metabolite list consisted exclusively of the differential metabolites (44 unique metabolites for spermatozoa and 9 unique metabolites for seminal plasma) that met the stringent criteria of FDR < 0.05, |log_2_FC| > 1, and VIP > 1. Multiple testing correction was performed using the Benjamini–Hochberg False Discovery Rate (FDR) procedure, and metabolic pathways were considered significantly enriched at FDR < 0.05.

## 3. Results

### 3.1. Analysis of Differential Metabolites in Spermatozoa Samples

Overall, a total of 249 unique metabolites were annotated based on the metabolomic analysis. In spermatozoa, 92 and 146 features (putatively annotated compounds) were identified in the negative and positive ionization modes, respectively, prior to stringent data filtering. Detailed information on feature counts at each stage of the data processing workflow is provided in Supplementary Table S3.

To assess the natural variation in the metabolomic profiles and validate the absence of analytical artifacts, unsupervised principal component analysis (PCA) was performed on the Pareto-scaled data prior to supervised modeling (Supplementary Figure S1).

In spermatozoa, the first principal component (PC1) explained 56.8% (negative mode) and 56.8% (positive mode) of the total variance. A clear trend of separation between HQ and LQ groups was observed along PC1 in both modes, with HQ samples predominantly clustering on the negative side and LQ samples on the positive side. The observed separation along PC1 indicates that variation associated with semen-quality grouping contributes substantially to the overall variance in the spermatozoa metabolomic profiles.

Visual inspection of PCA plots did not reveal extreme outliers, and the observed clustering was consistent with the predefined biological groups. However, because injection order, analytical batch, and pooled QC samples could not be formally assessed in this retrospective analysis, potential technical variation cannot be entirely ruled out, and the supervised analyses should be regarded as exploratory.

To visualize the comprehensive metabolomic profiles of the spermatozoa samples in both ionization modes, partial least squares-discriminant analysis (PLS-DA) score plots were constructed according to their assignment to the high-quality (HQ) and low-quality (LQ) groups (Figure 1).

The results of partial least squares-discriminant analysis (PLS-DA) of the spermatozoa metabolomic profiles in negative and positive ionization modes are presented. Each point on the score plot corresponds to an individual sample (*n* = 30). PLS-DA demonstrated clear separation between HQ and LQ groups in all datasets. Cross-validation identified one latent component as optimal for all datasets. The balanced error rate (BER) values were 0.147 for spermatozoa ESI+, 0.170 for spermatozoa ESI−, 0.210 for seminal plasma ESI+, and 0.190 for seminal plasma ESI−. The number of metabolites with stable VIP scores (>1.0 in at least 70% of bootstrap iterations) was 48 for spermatozoa ESI+, 25 for spermatozoa ESI−, 13 for seminal plasma ESI+, and 18 for seminal plasma ESI−. Permutation testing (200 permutations) confirmed that the observed discrimination significantly exceeded that expected by chance in all models (Spermatozoa ESI+, *p* = 0.005; Spermatozoa ESI−, *p* = 0.005; Seminal plasma ESI+, *p* = 0.015; Seminal plasma ESI−, *p* = 0.005). Teal and pink points represent the high-quality (HQ) and low-quality (LQ) sperm groups, respectively. The ellipses indicate the 68% confidence interval for each group, and the crosses (X) mark the centroids. A clear separation between the HQ and LQ groups is observed along the first component in both the negative (30.5% explained variance) and positive (23.3%) ionization modes, indicating substantial differences in the metabolite profiles of the sperm fraction between HQ and LQ bulls (Figure 1).

The volcano plots display significantly differential metabolites meeting the criteria of FDR < 0.05 and |log_2_FC| > 1. Both upregulated and downregulated metabolites were identified in the spermatozoa samples (Figure 2). The significantly differential metabolites detected in the spermatozoa samples are detailed in Table 1.

As shown in Table 1, a total of 46 LC–MS features corresponding to 44 unique metabolites (aspartic acid and glutamic acid were detected in both ionization modes) significantly differed between the LQ and HQ groups, meeting the selection criteria (FDR < 0.05, |log_2_FC| > 1, VIP > 1, and AUC ≤ 0.20 or ≥0.80). The majority of these metabolites belonged to the superclass of organic acids and derivatives (24 features/22 unique compounds), indicating substantial differences in amino acid and energy metabolism between the studied groups.

The most pronounced increase in the HQ group was observed for histidine (log_2_FC = 10.91), aspartic acid (log_2_FC = 10.38), pipecolic acid (log_2_FC = 7.76), lysine (log_2_FC = 7.55), delta-hydroxylysine (log_2_FC = 6.49), ornithine (log_2_FC = 6.33), and glutamic acid (log_2_FC = 6.24). Additionally, elevated levels of arginine, citrulline, glutamine, serine, and threonine were detected in the spermatozoa of the HQ bulls, indicating alterations in amino acid metabolism. The following compounds were elevated in the LQ group: spermidine (log_2_FC = −7.75), enterolactone (log_2_FC = −4.38), galacturonic acid (log_2_FC = −3.90), luteolin (log_2_FC = −3.49), 2′,6′-dihydroxy-4′-methoxyacetophenone (log_2_FC = −3.23), and nicotinic acid (log_2_FC = −3.20).

To facilitate interpretation of the group differences underlying the statistical prioritization, box plots of the PQN-normalized, log_2_-transformed peak intensities for the differential metabolites are provided in Supplementary Figure S2 (i.e., the same values that were used for statistical testing). For most metabolites, a clear separation between the HQ and LQ groups is evident on the normalized scale, consistent with the reported fold changes, AUC values, and VIP scores. Nevertheless, because several metabolites were close to the detection limit in one of the groups, the corresponding fold changes should be interpreted with appropriate caution.

The highest apparent discriminatory power was demonstrated by 7,8-dihydrobiopterin (AUC = 0.942, 95% CI: 0.836–1.000), glutamic acid (AUC = 0.933, 95% CI: 0.840–1.000), aconitic acid (AUC = 0.916, 95% CI: 0.796–0.987), 2′-deoxyguanosine 5′-monophosphate (AUC = 0.916, 95% CI: 0.778–1.000), mannitol (AUC = 0.902, 95% CI: 0.773–0.991), and glutamine (AUC = 0.889, 95% CI: 0.764–0.982). High VIP values for nicotinic acid (1.980), 7,8-dihydrobiopterin (1.861), 2′-deoxyguanosine 5′-monophosphate (1.730), glutamic acid (1.686), and aconitic acid (1.589) indicate their substantial contribution to group separation in the PLS-DA model.

For the differential metabolites in spermatozoa, we performed KEGG pathway enrichment analysis (Figure 3).

The results of the KEGG pathway enrichment analysis revealed that the differential metabolites in the spermatozoa of bulls with varying semen quality are predominantly involved in amino acid and nitrogen metabolism (Figure 3).

The most strongly enriched pathway was arginine biosynthesis, followed by D-amino acid metabolism, glutathione metabolism, lysine degradation, nicotinate and nicotinamide metabolism, histidine metabolism, arginine and proline metabolism, nitrogen metabolism, and beta-alanine metabolism. These pathways are unified by the central role of amino acids in protein biosynthesis, the maintenance of redox homeostasis, and cellular energy metabolism.

Collectively, these findings indicate that high semen quality is associated with profound rearrangements in amino acid metabolism and the antioxidant defense systems of spermatozoa.

The predominance of pathways associated with arginine, glutamate, and serine metabolism highlights the potential importance of nitrogen metabolism, antioxidant synthesis, and the maintenance of energy homeostasis in spermatozoa with high quality parameters.

The significantly enriched KEGG metabolic pathways for the differential metabolites in Holstein bull spermatozoa are presented in Table 2.

KEGG enrichment analysis of the differential metabolites in spermatozoa identified 11 statistically significant metabolic pathways (FDR < 0.05) (Table 2). The most pronounced enrichment was observed for the arginine biosynthesis pathway (FDR = 3.87 × 10^−6^), which included glutamic acid, arginine, citrulline, ornithine, aspartic acid and glutamine. Significant enrichment was also detected for D-amino acid metabolism, glutathione metabolism, lysine degradation, nicotinate and nicotinamide metabolism, histidine metabolism, arginine and proline metabolism, nitrogen metabolism, and beta-alanine metabolism, the latter linking aspartate, histidine, and spermidine to polyamine and redox-related metabolism. In addition, the updated differential metabolite list yielded significant enrichment of glycine, serine and threonine metabolism and glyoxylate and dicarboxylate metabolism, reflecting the contribution of serine, glycine, cystathionine, and threonine to the metabolic signature of semen quality. The glutathione metabolism pathway now also included pyroglutamic acid and spermidine in addition to glutamic acid and ornithine. Collectively, these findings suggest the potential involvement of altered amino acid, nitrogen, and dicarboxylate metabolism in bulls with high semen quality. However, because pathway enrichment is based on a limited number of annotated metabolites, these results should be interpreted as hypothesis-generating indicators of metabolic shifts rather than definitive confirmation of specific biochemical mechanisms.

### 3.2. Analysis of Differential Metabolites in Seminal Plasma Samples

Based on the metabolomic analysis, 113 and 121 features (putatively annotated metabolites) were putatively annotated in the seminal plasma in the negative and positive ionization modes, respectively, prior to stringent data filtering. To assess the natural variation in the metabolomic profiles and validate the absence of analytical artifacts, unsupervised principal component analysis (PCA) was performed on the Pareto-scaled data prior to supervised modeling (Supplementary Figure S1).

In seminal plasma, PC1 explained 30.7% (negative mode) and 28.1% (positive mode) of the variance. While the separation was less pronounced compared to spermatozoa, a distinct clustering trend was still evident, consistent with the weaker discrimination observed in subsequent PLS-DA analysis. This suggests that the seminal plasma metabolome is more heterogeneous and influenced by additional factors beyond semen quality classification.

The results of the partial least squares-discriminant analysis (PLS-DA) of seminal plasma metabolomic profiles in positive and negative ionization modes are presented. The HQ and LQ groups demonstrate separation along the first component in both ionization modes (16.9% for positive and 19.1% for negative ionization); however, the degree of separation is less pronounced compared to that of spermatozoa (Figure 4).

The volcano plot displays significantly differential metabolites meeting the criteria of FDR < 0.05 and |log_2_FC| > 1. Both upregulated and downregulated metabolites putatively annotated in the seminal plasma samples (Figure 5).

Significantly fewer differential metabolites were identified in the seminal plasma samples compared to the spermatozoa samples (Figure 5). Similar to the cellular samples, both upregulated and downregulated metabolites were observed.

Significantly fewer differential metabolites were identified in the seminal plasma samples compared to the spermatozoa samples (Figure 5). Similar to the cellular samples, both upregulated and downregulated metabolites were observed. In the seminal plasma of Holstein bulls, 11 differential LC–MS features corresponding to 9 unique metabolites (proline and indole-3-carboxyaldehyde were detected in both ionization modes) were identified between the HQ and LQ groups (Table 3). The majority of the identified compounds belonged to the superclass of organic acids and derivatives, which included amino acids, their derivatives, and intermediate metabolites of central metabolism.

In the HQ group, elevated levels of succinic acid (log_2_FC = 2.89), a tricarboxylic acid cycle intermediate, phenylalanine (log_2_FC = 1.81), and glutamine (log_2_FC = 1.80) were observed. Conversely, the LQ group exhibited elevated levels of 3-(4-hydroxyphenyl)lactic acid (log_2_FC = −2.17), indole-3-carboxyaldehyde (log_2_FC = −1.57 in ESI− and −1.25 in ESI+), isofraxidin (log_2_FC = −1.39), indolacetic acid (log_2_FC = −1.34), proline (log_2_FC = −1.21 in ESI− and −1.19 in ESI+), and ketoleucine (log_2_FC = −1.14). The most pronounced decrease in the HQ group was recorded for indole metabolites associated with tryptophan metabolism.

The highest apparent discriminatory power in seminal plasma was demonstrated by indole-3-carboxyaldehyde (AUC = 0.044, 95% CI: 0.000–0.124 in ESI+; AUC = 0.098, 95% CI: 0.009–0.222 in ESI−), ketoleucine (AUC = 0.102, 95% CI: 0.018–0.227), proline (AUC = 0.111, 95% CI: 0.004–0.262 in ESI+; AUC = 0.151, 95% CI: 0.027–0.324 in ESI−), indolacetic acid (AUC = 0.116, 95% CI: 0.004–0.271), glutamine (AUC = 0.862, 95% CI: 0.689–0.996), phenylalanine (AUC = 0.800, 95% CI: 0.613–0.947), and succinic acid (AUC = 0.831, 95% CI: 0.667–0.956). High VIP values for indole-3-carboxyaldehyde (2.409 in ESI+), proline (2.043 in ESI+), ketoleucine (1.932), indolacetic acid (1.790), and indole-3-carboxyaldehyde (1.758 in ESI−) indicate their substantial contribution to group separation in the PLS-DA model.

Notably, proline and indole-3-carboxaldehyde demonstrated consistent directional changes regardless of the ionization mode, further confirming the reliability of their annotation and the differences between the studied groups.

KEGG pathway enrichment analysis did not reveal any statistically significant metabolic pathways after correction for multiple testing (FDR > 0.05).

## 4. Discussion

Untargeted metabolomic profiling revealed distinct metabolic differences between Holstein bulls with high-quality (HQ) and low-quality (LQ) semen in both spermatozoa and seminal plasma. The most pronounced alterations were observed in pathways related to amino acid metabolism, energy metabolism, antioxidant defense, lipid metabolism, and metabolites potentially associated with the rumen microbiome and dietary components. The present study is exploratory in nature, and all mechanistic interpretations should be considered hypothesis-generating rather than confirmatory.

Given the exploratory nature of this study and the sample size, we have prioritized the discussion of the most statistically robust and biologically coherent candidate metabolites—specifically those strongly supported by KEGG pathway enrichment (e.g., glutamine, glutamate, aspartate, arginine, ornithine, citrulline, and succinate). The remaining differential metabolites identified in this analysis are presented as exploratory findings that provide valuable hypotheses for future targeted validation in larger cohorts.

Energy and carbohydrate metabolism. Spermatozoa from HQ bulls showed higher levels of energy metabolism-related metabolites, including 2′-deoxyguanosine 5′-monophosphate, aspartic acid, glutamic acid, and aconitic acid, a TCA cycle intermediate. Together, these metabolites indicate enhanced nucleotide turnover, amino acid metabolism, and mitochondrial energy production, processes that support ATP generation required for normal sperm function. ATP is also the immediate precursor for cyclic adenosine monophosphate (cAMP) synthesis, and adequate cellular energy metabolism is essential for maintaining cAMP-dependent signaling pathways involved in sperm capacitation and motility [29]. In humans, aspartic acid has been positively associated with normal sperm morphology, with reduced levels reported in teratozoospermia [30]. Similarly, reduced aconitic acid levels have been observed in men with reproductive disorders accompanied by impaired sperm energy metabolism [31]. In line with enhanced nucleotide turnover, 2′-deoxyguanosine 5′-monophosphate—a purine metabolite whose deficiency has been associated with impaired germ cell development [32]—was more abundant in spermatozoa from HQ bulls. Elevated succinate in seminal plasma from HQ bulls further supports this pattern; reduced succinate has been reported in human asthenozoospermia [33], and succinate supplementation has been shown to alleviate oxidative stress-induced sperm damage in boars [34]. However, because mitochondrial activity was not directly assessed in the present study, these observations should be interpreted as suggestive rather than definitive. Additionally, HQ bulls showed higher levels of mannitol and sorbitol, intermediates of the polyol pathway of glucose metabolism that reflect the intensity of carbohydrate metabolism [35]; their reduced abundance in the LQ group may indicate less efficient carbohydrate utilization and energy supply in spermatozoa, although this remains speculative.

Membrane-associated metabolites. Higher levels of N-acetyl-mannosamine were observed in spermatozoa from HQ bulls (log_2_FC = 3.71). N-acetyl-mannosamine is an amino sugar involved in glycoprotein and glycolipid biosynthesis and may contribute to membrane glycosylation patterns relevant to sperm function. The biological significance of this finding in the context of semen quality requires further investigation.

Amino acid and glutathione metabolism. KEGG pathway enrichment identified significant enrichment of arginine biosynthesis, D-amino acid metabolism, glutathione metabolism, lysine degradation, nicotinate and nicotinamide metabolism, histidine metabolism, arginine and proline metabolism, nitrogen metabolism, and beta-alanine metabolism, highlighting amino acid metabolism as a principal feature distinguishing the groups.

In particular, beta-alanine metabolism emerged as significantly enriched, since beta-alanine is a rate-limiting precursor for the synthesis of carnosine, a dipeptide with potent antioxidant and pH-buffering properties that protects spermatozoa against lipid peroxidation and oxidative stress. The intersection of beta-alanine metabolism with histidine and aspartate networks further supports the prominence of coordinated redox-related and amino acid rearrangements associated with high semen quality.

Histidine levels were higher in spermatozoa from HQ bulls. Altered histidine metabolism has been associated with male reproductive disorders, with increased histidine reported in a rat infertility model [36] and negatively correlated with sperm morphology in humans [37]. The apparent discrepancy with the present findings may reflect species-specific differences, or alternatively histidine may contribute to semen quality through its role as a precursor of carnosine, a dipeptide with antioxidant properties that may protect against lipid peroxidation.

Glutamine and glutamic acid were among the most consistent metabolites, elevated in HQ bulls in both spermatozoa and seminal plasma. These amino acids support energy production, mitochondrial function, nitrogen metabolism, and antioxidant defense. Reduced relative abundances of glutamate and glutamine have been associated with obesity, asthenozoospermia, and other male reproductive disorders in various species [38,39]. Glutamic acid has been reported to influence sperm function [40], and its deficiency has been linked to impaired spermatogenesis and male infertility in humans [41,42]. Glutamate and aspartate have also been shown to reduce oxidative stress in the testes and epididymis of boars, with their relative abundances positively associated with sperm quality [43]. Glutamine is a known precursor of glutathione; in bulls, supplementation improves cryotolerance by enhancing post-thaw motility, membrane and acrosome integrity, increasing SOD, GSH-Px, and CAT activities, and reducing MDA accumulation, while also supporting mitochondrial function through increased membrane potential and AMPK phosphorylation [44]. The pronounced increase in spermatozoa suggests glutamine may represent a candidate metabolite associated with high semen quality, though validation in independent populations is required.

Pyroglutamic acid, an intermediate of glutathione metabolism, was also differential. It has been detected in human seminal plasma in the context of recurrent spontaneous abortion [45], whereas Roberts et al. reported a tendency toward higher pyroglutamic acid in bulls with low semen quality [46]. These observations highlight the need for further investigation.

Serine, hypotaurine, cystathionine—all involved in transsulfuration, one-carbon metabolism, and glutathione biosynthesis—also differed between groups. Serine serves as a precursor for glycine and cysteine synthesis and participates in one-carbon metabolism essential for antioxidant defense and sperm maturation; it has also been implicated in sperm function in other mammals [47], while disturbances in serine and glycine metabolism have been reported in human oligozoospermia [48]. Hypotaurine is a well-recognized antioxidant; reduced levels have been reported in bulls with low semen quality [49], and low levels in human spermatozoa may reflect increased oxidative stress due to oxidation to taurine [50]. Impaired cystathionine metabolism has been linked to disrupted spermatogenesis in mouse models of folate and B12 deficiency [51]. Collectively, these findings suggest coordinated alterations in sulfur amino acid and redox-related metabolism associated with semen quality, although the specific mechanisms remain to be elucidated.

It is important to emphasize that the exceptionally large log_2_ fold changes observed for certain individual candidate metabolites, such as histidine (10.91), aspartic acid (10.38), and spermidine (−7.75), may be partly driven by their abundances approaching the lower limit of detection in the LQ group. Therefore, these large effect sizes should be interpreted with appropriate caution.

Arginine metabolism. HQ bulls had higher levels of ornithine, arginine, and citrulline, consistent with arginine biosynthesis as the most significantly enriched pathway. Reduced ornithine has been reported in human teratozoospermia and positively associated with sperm morphology [30]. Arginine is known to serve as a precursor of nitric oxide, which has been implicated in the regulation of sperm motility and intracellular signaling [52]. Disruption of arginine biosynthesis impairs Sertoli cell function and is accompanied by reduced citrulline; in sheep, citrulline supplementation has been shown to alleviate DNA damage in Sertoli cells and improve blood–testis barrier integrity [53]. The coordinated increase in these metabolites suggests enhanced arginine metabolism in HQ bulls, though nitric oxide metabolism and Sertoli cell function were not investigated in the present study.

Notably, two metabolites functionally linked to nitric oxide biology were also elevated in spermatozoa from HQ bulls: 7,8-dihydrobiopterin (log_2_FC = 2.75), the oxidized form of tetrahydrobiopterin (BH4), an essential cofactor of nitric oxide synthases, and SDMA (log_2_FC = 4.51), a methylated arginine derivative involved in the modulation of arginine–nitric oxide metabolism. Although nitric oxide signaling was not directly assessed, the coordinated increase in arginine, ornithine, citrulline, 7,8-dihydrobiopterin, and SDMA is consistent with altered activity of the arginine–NO axis in bulls with higher semen quality.

Threonine, an essential amino acid and precursor for glycine synthesis, was also elevated in spermatozoa from HQ bulls (log_2_FC = 3.72), consistent with the enrichment of glycine, serine and threonine metabolism and supporting the importance of one-carbon metabolism and glutathione biosynthesis in maintaining redox homeostasis. Notably, lysine (log_2_FC = 7.55) and several metabolites of its catabolism—pipecolic acid (log_2_FC = 7.76), delta-hydroxylysine (log_2_FC = 6.49), and saccharopine (log_2_FC = 5.48)—were elevated in spermatozoa from HQ bulls, consistent with the enrichment of lysine degradation and suggesting enhanced amino acid turnover and nitrogen metabolism in bulls with higher semen quality.

In seminal plasma, HQ bulls showed higher levels of phenylalanine (log_2_FC = 1.81), an essential aromatic amino acid and precursor of tyrosine, and of proline (log_2_FC = −1.21 in ESI− and −1.19 in ESI+), an amino acid implicated in osmoregulation, reactive oxygen species scavenging, and arginine/proline metabolism. Conversely, the LQ group exhibited elevated ketoleucine (log_2_FC = −1.14), a leucine catabolite, and 3-(4-hydroxyphenyl)lactic acid (log_2_FC = −2.17), a phenolic acid derived from aromatic amino acid metabolism, further supporting rearrangements of amino acid metabolism associated with semen quality. Lipid metabolism. Spermatozoa from HQ bulls showed increased sn-glycero-3-phosphocholine, a glycerophospholipid involved in membrane phospholipid turnover. Additionally, octanoylcarnitine and lauroylcarnitine were elevated in the LQ group. Acylcarnitines participate in fatty acid transport into mitochondria, and their altered abundance may reflect differences in lipid oxidation capacity between groups. These findings suggest alterations in membrane lipid and fatty acid metabolism may characterize semen quality differences, although functional validation is required.

Microbiota- and diet-associated metabolites. A subset of differential metabolites—including indoleacetic acid, indole-3-carboxaldehyde, tyramine, enterolactone, and several phenolic derivatives—has been previously associated with microbial transformation of dietary components in other biological systems. Indoleacetic acid has been proposed as a metabolite potentially linked to semen quality in boars [54]; indole-3-carboxaldehyde, a product of tryptophan metabolism frequently generated through microbial pathways in the gut [55], has been positively associated with sperm mitochondrial membrane integrity in rams [56]. Tyramine has been reported to arise from microbial decarboxylation of tyrosine in some contexts [57,58], and while not directly linked to semen quality, tyrosine metabolism is associated with sperm motility in humans [59]. Urolithin A, a microbial metabolite of dietary ellagitannins known for its anti-inflammatory and mitophagy-modulating properties [60], was elevated in spermatozoa from LQ bulls (log_2_FC = −1.70), providing additional evidence of diet- and microbiota-related contributions to the semen metabolome. Importantly, the present study did not include microbiome characterization; therefore, the origin of these metabolites cannot be conclusively attributed to microbial activity, and their presence may alternatively reflect dietary composition or other factors. Any potential link to rumen microbial metabolism requires validation through integrated multi-omics approaches.

Several plant-derived phenolic compounds and phytoestrogens—including enterolactone, hispidulin-7-glucuronide, and isofraxidin—were more abundant in LQ bulls. Isofraxidin, a hydroxycoumarin derivative, along with other plant-derived phenolic compounds, has been reported to possess antioxidant and anti-inflammatory properties in various experimental systems [61,62,63], including protection against oxidative testicular damage via the Nrf2/HO-1 pathway. Their accumulation may represent a compensatory metabolic response associated with altered redox homeostasis rather than a direct cause of reduced semen quality. However, because oxidative stress was not directly assessed in the present study, these interpretations remain speculative.

Metabolites elevated in LQ bulls. Higher levels of nicotinic acid, xanthurenic acid, and spermidine were observed in the LQ group. Oxidative stress is associated with cellular damage [64] and impaired male reproductive function [65], and has been shown to alter the metabolomic profile in various models, affecting metabolites such as creatine, carnitine, choline, and xanthurenic acid [66]. Nicotinic acid contributes to mitochondrial metabolism and redox-related pathways; mitochondria are the primary source of ATP for sperm motility and function in mammals [67]. Spermidine is involved in spermatogenesis and has been associated with germ cell development and sperm motility [68]. However, because oxidative stress markers were not directly measured, these interpretations remain hypothesis-generating.

Metabolic crosstalk between spermatozoa and seminal plasma. The integration of metabolomic data from both biological matrices highlights a fundamental metabolic crosstalk that dictates semen quality. While the sperm metabolome reflects the intrinsic cellular capacity for energy production, nucleotide turnover, and structural maintenance (e.g., amino acid metabolism), the seminal plasma metabolome acts as a critical extracellular buffer. Metabolites in the seminal plasma, including antioxidants, TCA cycle intermediates (e.g., succinate), and microbial-derived compounds, provide essential substrates and redox protection that sustain sperm viability and motility. Our findings suggest that alterations in the seminal plasma microenvironment, such as the accumulation of microbial metabolites or oxidative stress markers in LQ bulls, directly constrain the metabolic efficiency of the spermatozoa. This highlights that semen quality is not determined by the sperm cell in isolation, but by the synergistic metabolic integration of both matrices.

Several limitations should be considered. First, the sample size (*n* = 30) was relatively small; findings require validation in larger, independent populations. Second, sperm metabolite extraction was performed using a fixed volume (100 μL) without prior normalization to sperm cell number or protein content. Notably, sperm concentration was significantly higher in the HQ group (1.66 ± 0.15 × 10^9^ cells/mL) compared to the LQ group (1.49 ± 0.25 × 10^9^ cells/mL, *p* = 0.04; Supplementary Table S1). Therefore, while Probabilistic Quotient Normalization (PQN) was applied during preprocessing to account for general dilution effects, we cannot definitively rule out that some of the observed differences in metabolite abundance reflect variations in the total cellular biomass extracted per fixed volume, rather than true changes in metabolite concentration per individual cell. Third, spermatozoa were not washed before extraction to preserve the endogenous metabolomic profile, which may have resulted in minor residual seminal plasma contamination. Fourth, preprocessing procedures including imputation, normalization, transformation, and scaling were applied before cross-validation in the PLS-DA workflow; while commonly applied in exploratory studies, this may introduce some information leakage, which should be considered when interpreting the reported Balanced Error Rate (BER) values and the apparent model performance. Fifth, regarding analytical quality control, the internal standards used in this study (amitriptyline and verapamil) were employed solely for monitoring extraction consistency and instrument performance, not for normalization of metabolite abundances. These two compounds cannot represent the broad chemical diversity of the detected metabolome, and the absence of class-specific or isotopically labeled internal standards limits the ability to correct for compound-specific matrix effects or ionization differences. Sixth, several advanced QC procedures (e.g., QC-based feature filtering using relative standard deviation, mathematical signal drift correction, and PCA clustering of pooled QC samples) were not performed, as the specific raw data for pooled QC injections were not retained for this retrospective subset analysis. Therefore, the supervised analyses should be regarded as exploratory. Seventh, classification was based on semen quality characteristics and ejaculate rejection rates rather than field fertility outcomes; the relationship with actual fertility remains to be established. Eighth, while PLS-DA component selection utilized cross-validation, univariate statistical metrics (fold changes, *p*-values, FDR) and AUC values were calculated using the full exploratory dataset; bootstrap resampling was used to estimate AUC confidence intervals, but these metrics do not represent externally validated predictive performance. Ninth, KEGG pathway enrichment was based on a limited number of annotated metabolites (particularly in seminal plasma, where only 9 differential metabolites were identified) and should be interpreted with caution. Tenth, although all bulls were maintained under identical housing, feeding, and management conditions, the rumen microbiome was not directly characterized; therefore, individual microbiome variations remain a potential unmeasured confounder for metabolites potentially originating from microbial transformation, and oxidative stress was inferred from metabolomic patterns rather than direct biochemical measurements. Finally, because this study lacked an independent validation cohort, the identified metabolites should be strictly described as candidate metabolites associated with semen-quality classification that need confirmation in a larger independent population, rather than validated biomarkers.

## 5. Conclusions

In summary, this exploratory study detected a range of metabolites that differ between Holstein bulls with high- and low-quality semen, particularly in pathways related to amino acid metabolism, energy metabolism, redox homeostasis, and membrane lipid composition. These findings should be viewed as hypothesis-generating rather than definitive. The observed metabolic signatures suggest that high semen quality in bulls may be associated with more efficient energy metabolism, mitochondrial activity, and antioxidant defense, whereas the LQ group was characterized by the accumulation of metabolites potentially reflecting compensatory responses to metabolic and oxidative stress. The most promising candidate metabolites detected in this exploratory study—including glutamine, glutamate, succinate, aspartate, ornithine, citrulline, and arginine—require validation in larger, independent populations with available field fertility records to establish their biological and practical significance.

## Figures and Tables

**Figure 1:**
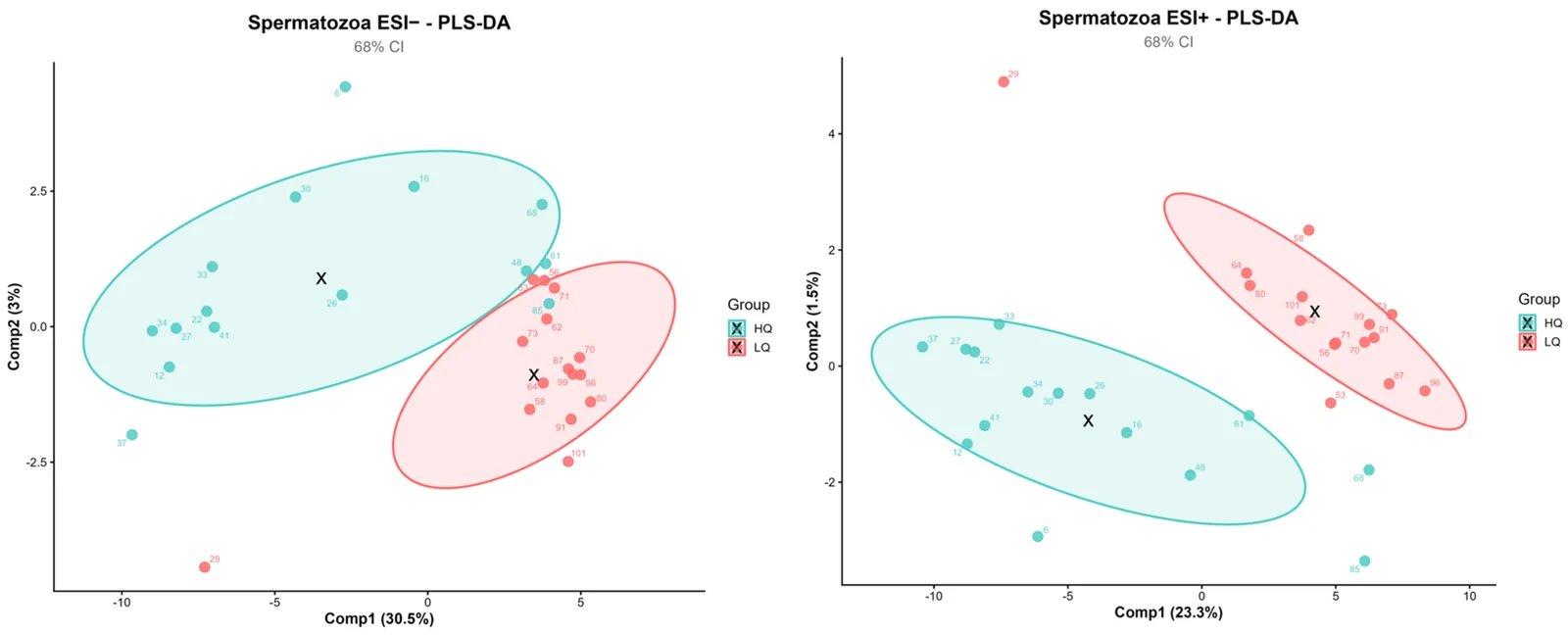
PLS-DA (partial least squares-discriminant analysis) of spermatozoa in negative (**left**) and positive (**right**) ionization modes. Although five-fold cross-validation identified one latent component as optimal for both spermatozoa datasets, two components are displayed for visualization purposes.

**Figure 2:**
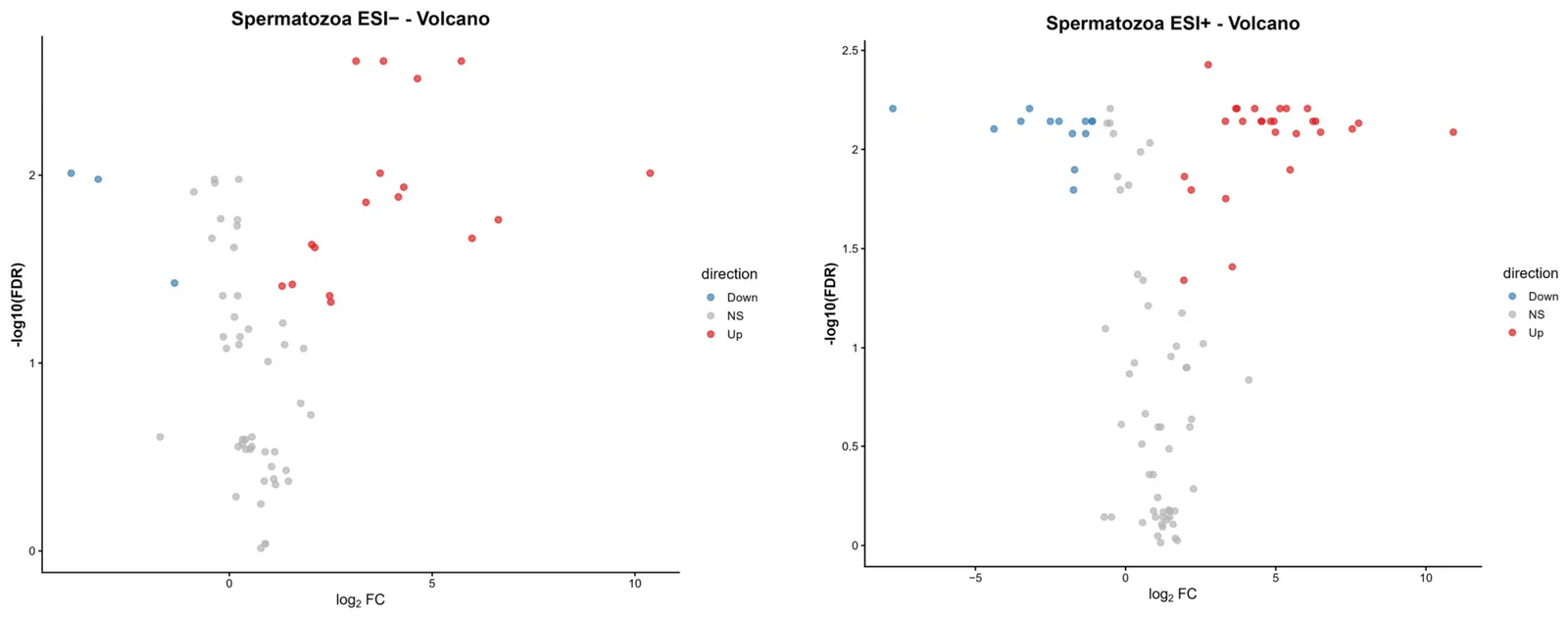
Volcano plots comparing metabolite levels in bull spermatozoa in negative (**left**) and positive (**right**) ionization modes.

**Figure 3:**
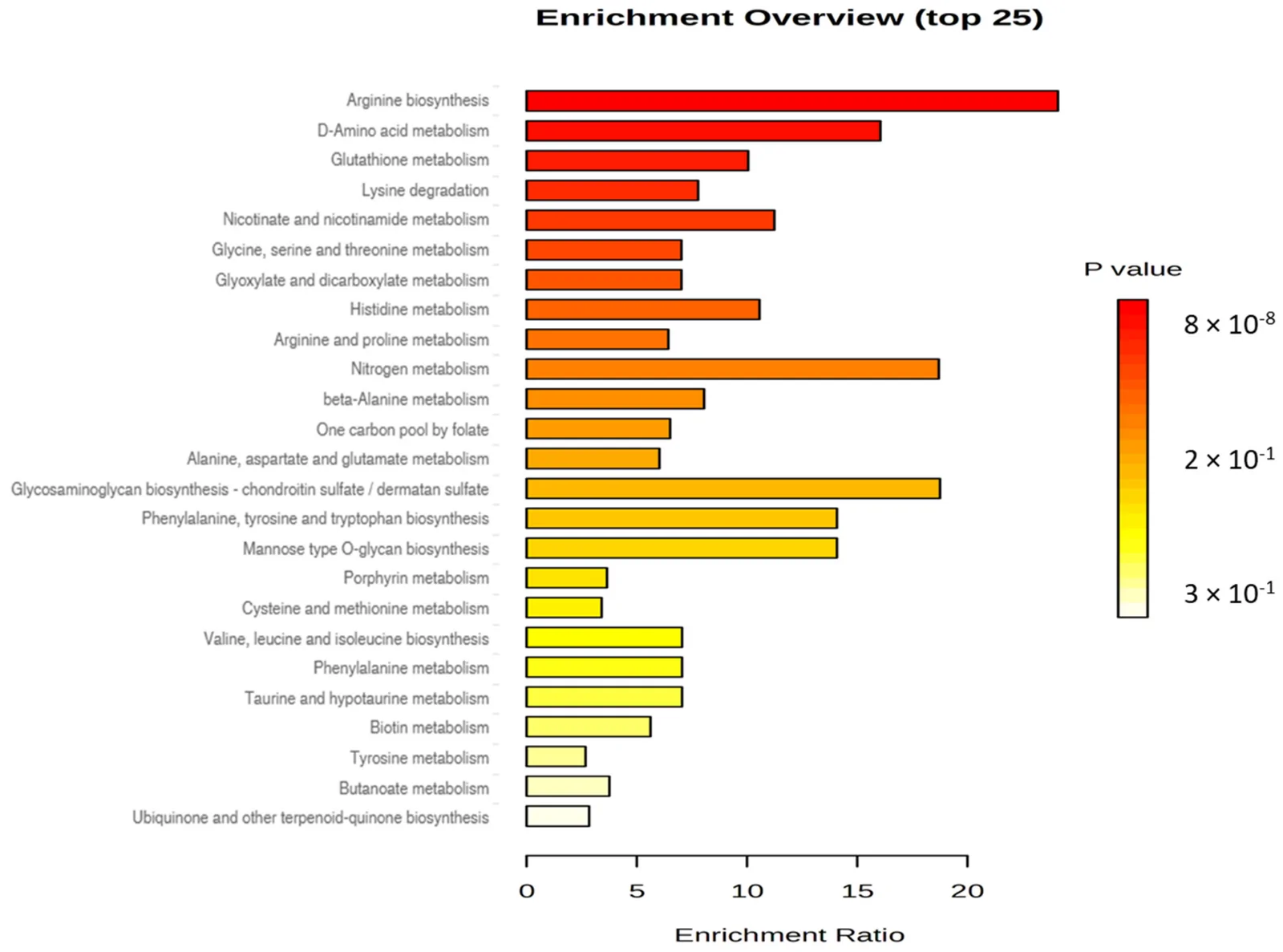
KEGG pathway enrichment analysis of the differential metabolites in Holstein bull spermatozoa (top 25). The length of the bars represents the enrichment ratio, while the color indicates the statistical significance (*p*-value). A more intense red color corresponds to lower *p*-values and higher statistical significance of the pathway.

**Figure 4:**
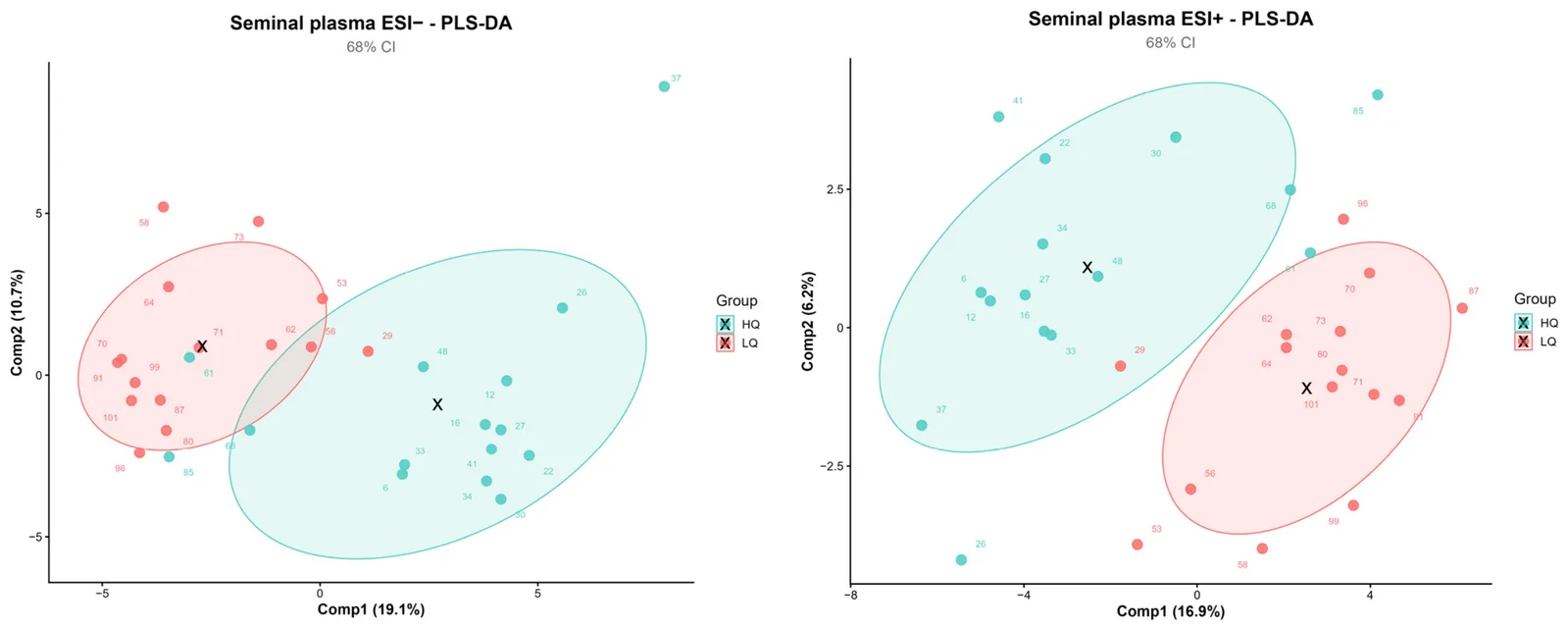
PLS-DA (partial least squares-discriminant analysis) of seminal plasma in negative (**left**) and positive (**right**) ionization modes. Although five-fold cross-validation identified one latent component as optimal for both seminal plasma datasets, two components are displayed for visualization purposes.

**Figure 5:**
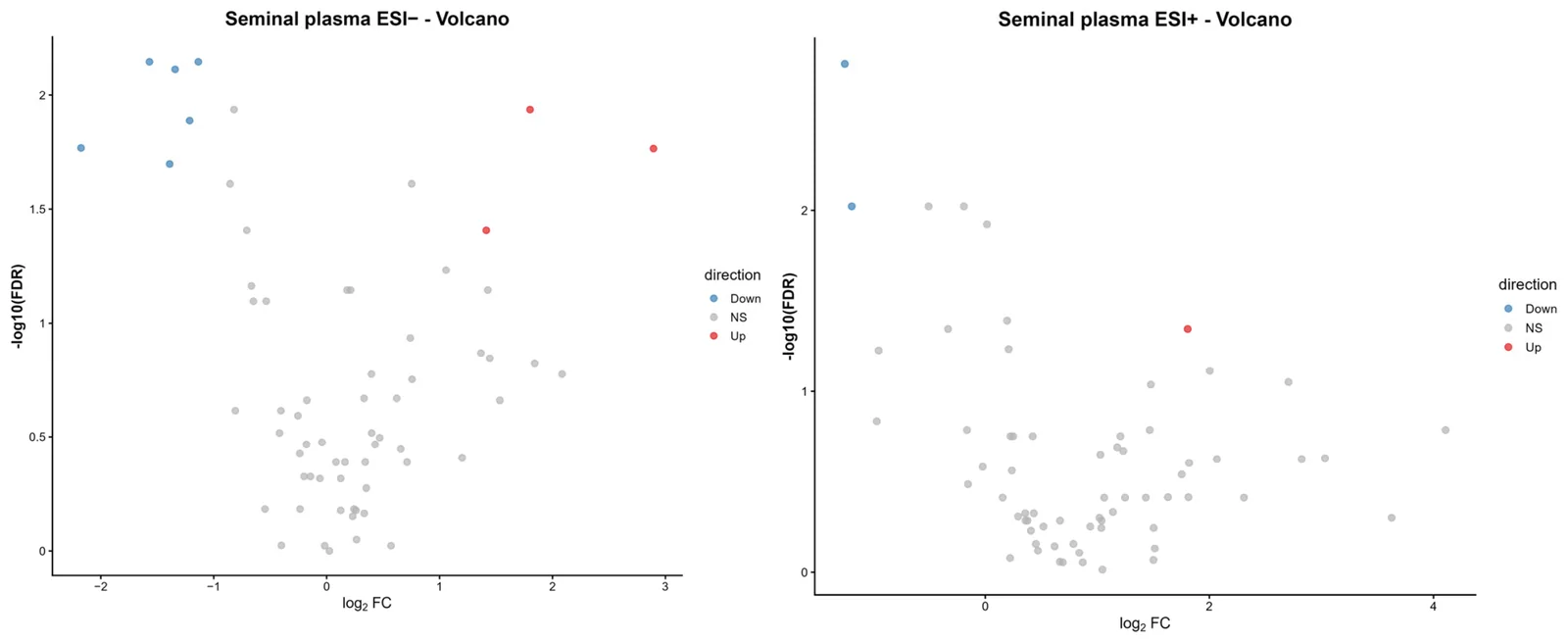
Volcano plots showing differential metabolite analysis in bull seminal plasma in negative (**left**) and positive (**right**) ionization modes.

**Table 1:** Differential metabolites in Holstein bull spermatozoa significantly differing between the HQ and LQ groups (FDR < 0.05, |log_2_FC| > 1, VIP > 1, and AUC ≤ 0.20 or ≥0.80). Metabolites are grouped by ClassyFire superclasses and ranked by the absolute value of log_2_FC.

Superclass	Metabolite	Mode	CID	log_2_FC	*p*	AUC (95% CI)	FDR	VIP
Organic acids and derivatives	Histidine	ESI+	6274	10.91	0.0028	0.822 (0.622–0.991)	0.0082	1.433
	**Aspartic acid**	ESI−	5960	10.38	0.0011	0.853 (0.689–0.969)	0.0098	1.479
	Pipecolic acid	ESI+	849	7.76	0.0021	0.831 (0.649–0.973)	0.0074	1.264
	Lysine	ESI+	5962	7.55	0.0025	0.827 (0.644–0.978)	0.0079	1.297
	Delta-hydroxylysine	ESI+	3032849	6.49	0.0028	0.822 (0.649–0.947)	0.0082	1.371
	Ornithine	ESI+	6262	6.33	0.0014	0.844 (0.698–0.964)	0.0072	1.351
	**Glutamic acid**	ESI+	33032	6.24	0.0012	0.849 (0.680–0.982)	0.0072	1.440
	Pyroglutamic acid	ESI+	7405	6.06	0.0007	0.867 (0.698–0.978)	0.0062	1.459
	**Glutamic acid**	ESI−	33032	5.72	0.00006	0.933 (0.840–1.000)	0.0025	1.686
	**Aspartic acid**	ESI+	5960	5.68	0.0032	0.818 (0.640–0.978)	0.0083	1.296
	Saccharopine	ESI+	160556	5.48	0.0055	0.800 (0.618–0.938)	0.0127	1.200
	Cystathionine	ESI+	834	5.35	0.0007	0.867 (0.716–0.969)	0.0062	1.405
	Serine	ESI+	5951	5.14	0.0005	0.876 (0.724–0.982)	0.0062	1.385
	Gly-Leu dipeptide	ESI+	92843	4.83	0.0012	0.849 (0.684–0.973)	0.0072	1.343
	Arginine	ESI+	6322	4.54	0.0019	0.836 (0.667–0.982)	0.0072	1.288
	SDMA	ESI+	169148	4.51	0.0009	0.858 (0.698–0.982)	0.0072	1.258
	Citrulline	ESI+	9750	4.30	0.0007	0.867 (0.711–0.982)	0.0062	1.447
	Glycine	ESI−	750	4.30	0.0021	0.831 (0.653–0.978)	0.0116	1.338
	Hypotaurine	ESI−	107812	4.17	0.0028	0.822 (0.631–0.973)	0.0123	1.312
	Galacturonic acid	ESI−	439218	−3.90	0.0011	0.147 (0.013–0.320)	0.0098	1.478
	Threonine	ESI−	6288	3.72	0.0009	0.858 (0.698–0.969)	0.0098	1.538
	Glutamine	ESI+	5961	3.67	0.0003	0.889 (0.764–0.982)	0.0062	1.480
	Tyrosine	ESI+	6057	3.32	0.0016	0.840 (0.676–0.982)	0.0072	1.292
	Aconitic acid	ESI−	309	3.12	0.0001	0.916 (0.796–0.987)	0.0025	1.589
Nucleosides, nucleotides, and analogues	N6-threonylcarbamoyladenosine	ESI+	161466	3.90	0.0016	0.840 (0.676–0.964)	0.0072	1.335
	2′-deoxyguanosine 5′-monophosphate	ESI−	135398597	3.80	0.0001	0.916 (0.778–1.000)	0.0025	1.730
Organic oxygen compounds	Phenethyl 2-O-(beta-D-xylopyranosyl)-beta-D-glucopyranoside	ESI−	24121293	6.63	0.0048	0.804 (0.613–0.965)	0.0173	1.267
	Mannitol	ESI−	6251	4.64	0.0002	0.902 (0.773–0.991)	0.0031	1.448
	N-acetyl-mannosamine	ESI+	439281	3.71	0.0004	0.884 (0.733–0.991)	0.0062	1.494
	Sorbitol	ESI−	5780	3.37	0.0032	0.818 (0.644–0.960)	0.0134	1.388
	2′,6′-dihydroxy-4′-methoxyacetophenone	ESI−	24135	−3.23	0.0014	0.156 (0.018–0.329)	0.0105	1.387
Organoheterocyclic compounds	Nicotinamide	ESI+	936	4.99	0.0028	0.822 (0.644–0.978)	0.0082	1.254
	Nicotinic acid	ESI+	938	−3.20	0.0002	0.102 (0.018–0.218)	0.0062	1.980
	7,8-dihydrobiopterin	ESI+	135398687	2.75	0.00004	0.942 (0.836–1.000)	0.0037	1.861
	Xanthurenic acid	ESI+	5699	−1.77	0.0032	0.182 (0.022–0.364)	0.0083	1.410
	Urolithin A	ESI+	5488186	−1.70	0.0055	0.200 (0.018–0.400)	0.0127	1.155
Phenylpropanoids and polyketides	Luteolin	ESI+	5280445	−3.49	0.0014	0.156 (0.009–0.329)	0.0072	1.342
	Hispidulin 7-O-glucuronide	ESI+	5318059	−2.51	0.0019	0.164 (0.018–0.342)	0.0072	1.187
	Chrysoeriol	ESI+	5280666	−1.34	0.0019	0.164 (0.018–0.347)	0.0072	1.195
	Calycosin	ESI+	5280448	−1.33	0.0032	0.182 (0.044–0.351)	0.0083	1.150
Benzenoids	Tyramine	ESI+	5610	−1.11	0.0019	0.164 (0.018–0.338)	0.0072	1.454
Lignans, neolignans and related compounds	Enterolactone	ESI+	114739	−4.38	0.0025	0.173 (0.031–0.351)	0.0079	1.394
	sn-glycero-3-phosphocholine	ESI+	657272	4.93	0.0012	0.849 (0.684–0.973)	0.0072	1.331
Lipids and lipid-like molecules	Octanoylcarnitine	ESI+	123701	−2.22	0.0019	0.164 (0.027–0.342)	0.0072	1.339
	Lauroylcarnitine	ESI+	168381	−1.12	0.0012	0.151 (0.027–0.311)	0.0072	1.331
Organic nitrogen compounds	Spermidine	ESI+	1102	−7.75	0.0007	0.133 (0.013–0.289)	0.0062	1.391

Note: Positive log_2_FC values indicate higher metabolite levels in the HQ group, whereas negative values indicate higher levels in the LQ group. ESI+, positive electrospray ionization; ESI−, negative electrospray ionization; AUC, area under the ROC curve; VIP, variable importance in projection in the PLS-DA model. All metabolites listed in this table met the stringent stability criterion, maintaining a VIP score > 1.0 in at least 70% of 100 bootstrap resampling iterations. Metabolites detected in both ionization modes are highlighted in bold. All metabolites were annotated at MSI confidence Level 2 (putative annotation) based on accurate mass, retention time, adduct assignment, isotope pattern, and MS/MS spectral matching against reference libraries, without confirmation using authentic reference standards. Detailed annotation information is provided in Supplementary Table S2.

**Table 2:** Significantly enriched KEGG metabolic pathways for the differential metabolites in Holstein bull spermatozoa.

KEGG Pathway	Total	Hits	Identified Metabolites	*p*-Value	FDR
Arginine biosynthesis	14	6	Glutamic acid; Arginine; Citrulline; Ornithine; Aspartic acid; Glutamine	4.78 × 10^−8^	3.87 × 10^−6^
D-Amino acid metabolism	14	4	Glutamine; Glutamic acid; Serine; Ornithine	7.04 × 10^−5^	0.00242
Glutathione metabolism	28	5	Glycine; Glutamic acid; Pyroglutamic acid; Ornithine; Spermidine	8.95 × 10^−5^	0.00242
Lysine degradation	29	4	Lysine; Saccharopine; Delta-hydroxylysine; Pipecolic acid	0.00139	0.0235
Nicotinate and nicotinamide metabolism	15	3	Aspartic acid; Nicotinic acid; Nicotinamide	0.00198	0.0235
Glycine, serine and threonine metabolism	32	4	Serine; Glycine; Cystathionine; Threonine	0.00203	0.0235
Glyoxylate and dicarboxylate metabolism	32	4	Serine; Glycine; Glutamic acid; Glutamine	0.00203	0.0235
Histidine metabolism	16	3	Glutamic acid; Histidine; Aspartic acid	0.00241	0.0244
Arginine and proline metabolism	35	4	Arginine; Ornithine; Spermidine; Glutamic acid	0.00285	0.0256
Nitrogen metabolism	6	2	Glutamine; Glutamic acid	0.00437	0.0354
beta-Alanine metabolism	21	3	Aspartic acid; Histidine; Spermidine	0.00538	0.0397

**Table 3:** Differential metabolites in the seminal plasma of Holstein bulls significantly differing between the HQ and LQ groups (FDR < 0.05, |log_2_FC| > 1, VIP > 1, and AUC ≤ 0.20 or ≥0.80). Metabolites are grouped by ClassyFire superclasses and ranked by the absolute value of log_2_FC.

Superclass	Metabolite	Ionization Mode	PubChem CID	log_2_FC	*p*-Value	AUC (95% CI)	FDR	VIP
	Succinic acid	ESI−	1110	2.89	0.0021	0.831 (0.667–0.956)	0.0172	1.655
	3-(4-hydroxyphenyl)lactic acid	ESI−	9378	−2.17	0.0019	0.164 (0.027–0.342)	0.0171	1.685
	Phenylalanine	ESI+	6140	1.81	0.0054	0.800 (0.613–0.947)	0.0450	1.649
Organic acids and derivatives	Glutamine	ESI−	5961	1.80	0.0008	0.862 (0.689–0.996)	0.0116	1.512
	**Proline**	ESI−	145742	−1.21	0.0012	0.151 (0.027–0.324)	0.0130	1.752
	**Proline**	ESI+	145742	−1.19	0.0004	0.111 (0.004–0.262)	0.0095	2.043
	Ketoleucine	ESI−	70	−1.14	0.0002	0.102 (0.018–0.227)	0.0071	1.932
Phenylpropanoids	Isofraxidin	ESI−	5318565	−1.39	0.0028	0.178 (0.044–0.346)	0.0201	1.560
	**Indole-3-carboxyaldehyde**	ESI−	10256	−1.57	0.0002	0.098 (0.009–0.222)	0.0071	1.758
Organoheterocyclic compounds	Indolacetic acid	ESI−	802	−1.34	0.0004	0.116 (0.004–0.271)	0.0077	1.790
	**Indole-3-carboxyaldehyde**	ESI+	10256	−1.25	2.33 × 10^−5^	0.044 (0.000–0.124)	0.0016	2.409

Note: Positive log_2_FC values indicate higher metabolite levels in the HQ group, whereas negative values indicate higher levels in the LQ group. ESI+, positive electrospray ionization; ESI−, negative electrospray ionization; AUC, area under the ROC curve; VIP, variable importance in projection in the PLS-DA model. All metabolites listed in this table met the stringent stability criterion, maintaining a VIP score > 1.0 in at least 70% of 100 bootstrap resampling iterations. Metabolites detected in both ionization modes are highlighted in bold. All metabolites were annotated at MSI confidence Level 2 (putative annotation) based on accurate mass, retention time, adduct assignment, isotope pattern, and MS/MS spectral matching against reference libraries, without confirmation using authentic reference standards. Detailed annotation information is provided in Supplementary Table S2. In the seminal plasma of Holstein bulls, 9 unique differential metabolites (11 LC–MS features) were identified between the HQ and LQ groups, meeting the statistical significance criteria (FDR < 0.05, |log_2_FC| > 1, VIP > 1, and AUC > 0.80 or <0.20). The majority of the identified compounds belonged to the superclass of organic acids and derivatives, which included amino acids, their derivatives, and intermediate metabolites of central metabolism.

## Data Availability

The raw mass spectrometry data, processed peak tables, sample metadata, and annotation tables generated in this study have been deposited in the MassIVE repository under the accession number MSV000102514 (DOI: 10.25345/C59Z90S4M).

## Supplementary Materials

The following supporting information can be downloaded at https://www.mdpi.com/article/10.3390/ani16193098/s1, Figure S1: Principal component analysis (PCA) score plots of spermatozoa and seminal plasma metabolomic profiles; Figure S2: Normalized peak intensities of the differential metabolites in spermatozoa and seminal plasma Metabolites meeting the significance criteria (FDR < 0.05, |log_2_FC| > 1, VIP > 1, VIP stability > 70% in bootstrap resampling, and AUC ≤ 0.20 or ≥ 0.80) are shown as box plots of log_2_-transformed intensities after Probabilistic Quotient Normalization (PQN), i.e., the same values that were used for statistical comparison. (A) Spermatozoa, ESI+ and (B) Spermatozoa, ESI−—the ten metabolites with the largest absolute log_2_FC in the corresponding ionization mode (the full lists of significant spermatozoa metabolites are provided in Table 1 and Supplementary Table S2); (C) Seminal plasma, ESI− (8 metabolites) and (D) Seminal plasma, ESI+ (3 metabolites)—all significant metabolites for the corresponding matrix and mode. Metabolites are ordered by decreasing |log_2_FC| within each panel; facet labels indicate the log_2_FC and FDR values. Box plots show the median (center line), interquartile range (box), and 1.5× IQR whiskers; individual data points (*n* = 15 per group) are overlaid as jittered dots. HQ = high-quality group; LQ = low-quality group; Table S1: Phenotypic characteristics and semen quality parameters of Holstein bulls included in the metabolomic study; Table S2: Annotation and quality-control information for metabolites detected in spermatozoa and seminal plasma by untargeted LC–MS analysis; Table S3: The number of features at each filtering step is as follows.

## Abbreviations

The following abbreviations are used in this manuscript:

AMPK	AMP-activated protein kinase
ATP	Adenosine triphosphate
AUC	Area Under the Curve
CAT	Catalase
cAMP	Cyclic adenosine monophosphate
ESI	Electrospray Ionization
FDR	False Discovery Rate
FC	Fold Change
GPx	Glutathione peroxidase
HILIC	Hydrophilic Interaction Liquid Chromatography
HQ	High Quality
KEGG	Kyoto Encyclopedia of Genes and Genomes
LC-MS/MS	Liquid Chromatography-Tandem Mass Spectrometry
LQ	Low Quality
LysoPC	Lysophosphatidylcholine
MDA	Malondialdehyde
MMP	Mitochondrial membrane potential
PLS-DA	Partial Least Squares Discriminant Analysis
PQN	Probabilistic Quotient Normalization
ROC	Receiver Operating Characteristic
SDMA	Symmetric Dimethylarginine
SOD	Superoxide Dismutase
VIP	Variable Importance in Projection
