# Supplementary material for: Untargeted Metabolomic Profiling of Spermatozoa and Seminal Plasma Identifies Candidate Metabolic Indicators of Semen Quality in Holstein Bulls
Source: Animals (Basel). 2026 Oct 2;16(19):3098. doi: 10.3390/ani16193098 (PMC13663586; doi:10.3390/ani16193098)
Supplement: Supplementary file 1 [file animals-16-03098-s001.zip › Supplementary_Figure_S1.pdf]

Supplementary Figure S1. Principal component analysis (PCA) score plots of spermatozoa and seminal plasma metabolomic profiles.

PCA was performed on Pareto-scaled data prior to supervised modeling to visualize the natural clustering of samples and to identify extreme outliers. (A) Spermatozoa, negative ionization mode (PC1: 56.8%, PC2: 7.3%). (B) Spermatozoa, positive ionization mode (PC1: 56.8%, PC2: 8.9%). (C) Seminal plasma, negative ionization mode (PC1: 30.7%, PC2: 16.9%). (D) Seminal plasma, positive ionization mode (PC1: 28.1%, PC2: 17.9%). Teal and red (salmon) points represent the high-quality (HQ) and low-quality (LQ) groups, respectively. Ellipses indicate the 68% confidence interval for each group. Crosses (X) mark the group centroids. Sample numbers are indicated for each point. The plots illustrate group-wise clustering trends and the absence of extreme outliers; because QC-based drift correction and PCA of pooled QC samples could not be performed (see Limitations), these plots are intended as descriptive visualization and should not be interpreted as formal evidence that analytical variability had no influence.

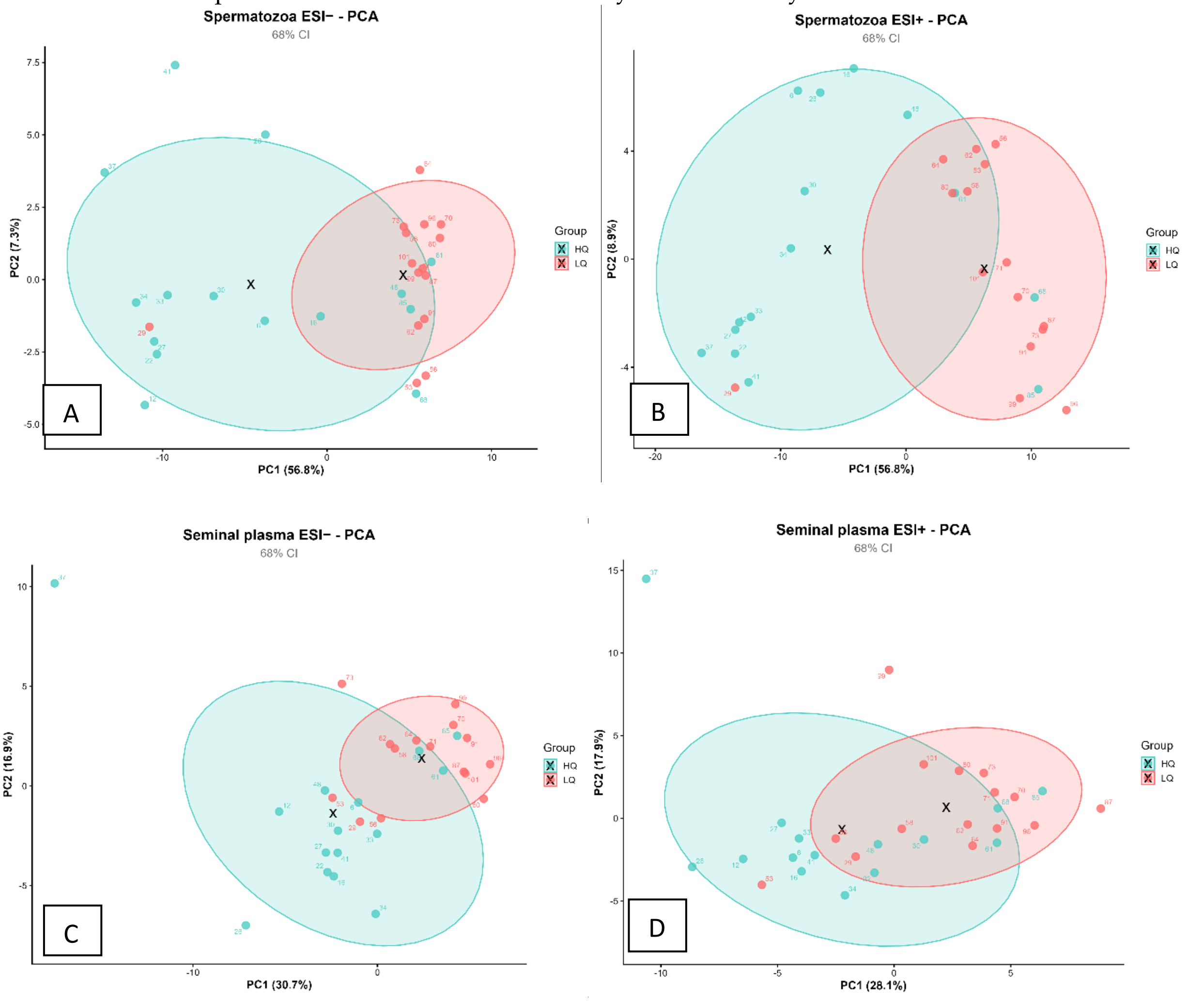

(A) Spermatozoa, negative ESI mode

Figure S1A. PCA score plot of spermatozoa metabolomic profile in negative ionization mode. PC1 explains 56.8% and PC2 explains 7.3% of the total variance. A separation trend between HQ and LQ groups is observed along PC1, indicating that metabolic differences associated with semen quality represent a notable source of biological variation.

(B) Spermatozoa, positive ESI mode

Figure S1B. PCA score plot of spermatozoa metabolomic profile in positive ionization mode. PC1 explains 56.8% and PC2 explains 8.9% of the total variance. Similar to the negative mode, a distinct clustering trend is evident along PC1, similar to the negative mode, a distinct clustering trend is evident along PC1, supporting the robustness of the observed metabolic differences.

(C) Seminal plasma, negative ESI mode

Figure S1C. PCA score plot of seminal plasma metabolomic profile in negative ionization mode. PC1 explains 30.7% and PC2 explains 16.9% of the total variance. While the separation is less pronounced compared with spermatozoa, a clustering trend is still evident, consistent with the greater heterogeneity of the seminal plasma metabolome.

(D) Seminal plasma, positive ESI mode

Figure S1D. PCA score plot of seminal plasma metabolomic profile in positive ionization mode. PC1 explains 28.1% and PC2 explains 17.9% of the total variance. The separation between groups is less distinct, suggesting that the seminal plasma metabolome is more heterogeneous and influenced by additional factors beyond semen quality classification.
