# Supplementary material for: Untargeted Metabolomic Profiling of Spermatozoa and Seminal Plasma Identifies Candidate Metabolic Indicators of Semen Quality in Holstein Bulls
Source: Animals (Basel). 2026 Oct 2;16(19):3098. doi: 10.3390/ani16193098 (PMC13663586; doi:10.3390/ani16193098)
Supplement: Supplementary file 1 [file animals-16-03098-s001.zip › Supplementary_Figure_S2.pdf]

Supplementary Figure S2. Normalized peak intensities of the differential metabolites in spermatozoa and seminal plasma. Metabolites meeting the significance criteria (FDR < 0.05, $|\log_2 FC| > 1$, VIP > 1, VIP stability > 70% in bootstrap resampling, and AUC ≤ 0.20 or ≥ 0.80) are shown as box plots of $\log_2$-transformed intensities after Probabilistic Quotient Normalization (PQN), i.e., the same values that were used for statistical comparison. (A) Spermatozoa, ESI+ and (B) Spermatozoa, ESI− — the ten metabolites with the largest absolute $\log_2$FC in the corresponding ionization mode (the full lists of significant spermatozoa metabolites are provided in Table 1 and Supplementary Table S2); (C) Seminal plasma, ESI− (8 metabolites) and (D) Seminal plasma, ESI+ (3 metabolites) — all significant metabolites for the corresponding matrix and mode. Metabolites are ordered by decreasing $|\log_2 FC|$ within each panel; facet labels indicate the $\log_2$FC and FDR values. Box plots show the median (center line), interquartile range (box), and 1.5× IQR whiskers; individual data points (n = 15 per group) are overlaid as jittered dots. HQ = high-quality group; LQ = low-quality group.

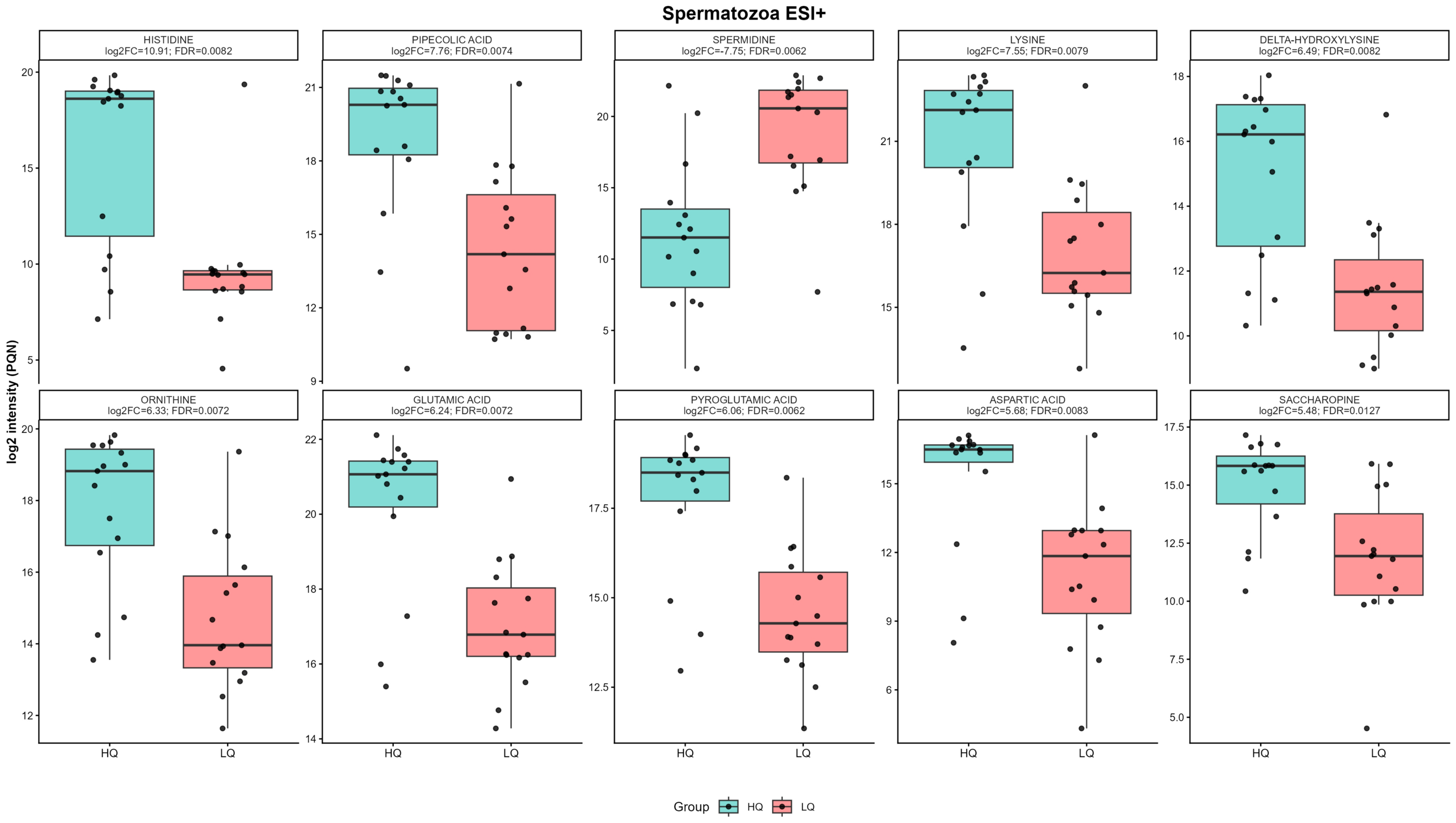
Spermatozoa ESI+
HISTIDINE
log2FC=10.91; FDR=0.0082
PIPECOLIC ACID
log2FC=7.76; FDR=0.0074
SPERMIDINE
log2FC=-7.75; FDR=0.0062
LYSINE
log2FC=7.55; FDR=0.0079
DELTA-HYDROXYLYSINE
log2FC=6.49; FDR=0.0082
ORNITHINE
log2FC=6.33; FDR=0.0072
GLUTAMIC ACID
log2FC=6.24; FDR=0.0072
PYROGLUTAMIC ACID
log2FC=6.06; FDR=0.0062
ASPARTIC ACID
log2FC=5.68; FDR=0.0083
SACCHAROPINE
log2FC=5.48; FDR=0.0127
log2 intensity (PQN)
HQ
LQ
Group HQ LQ

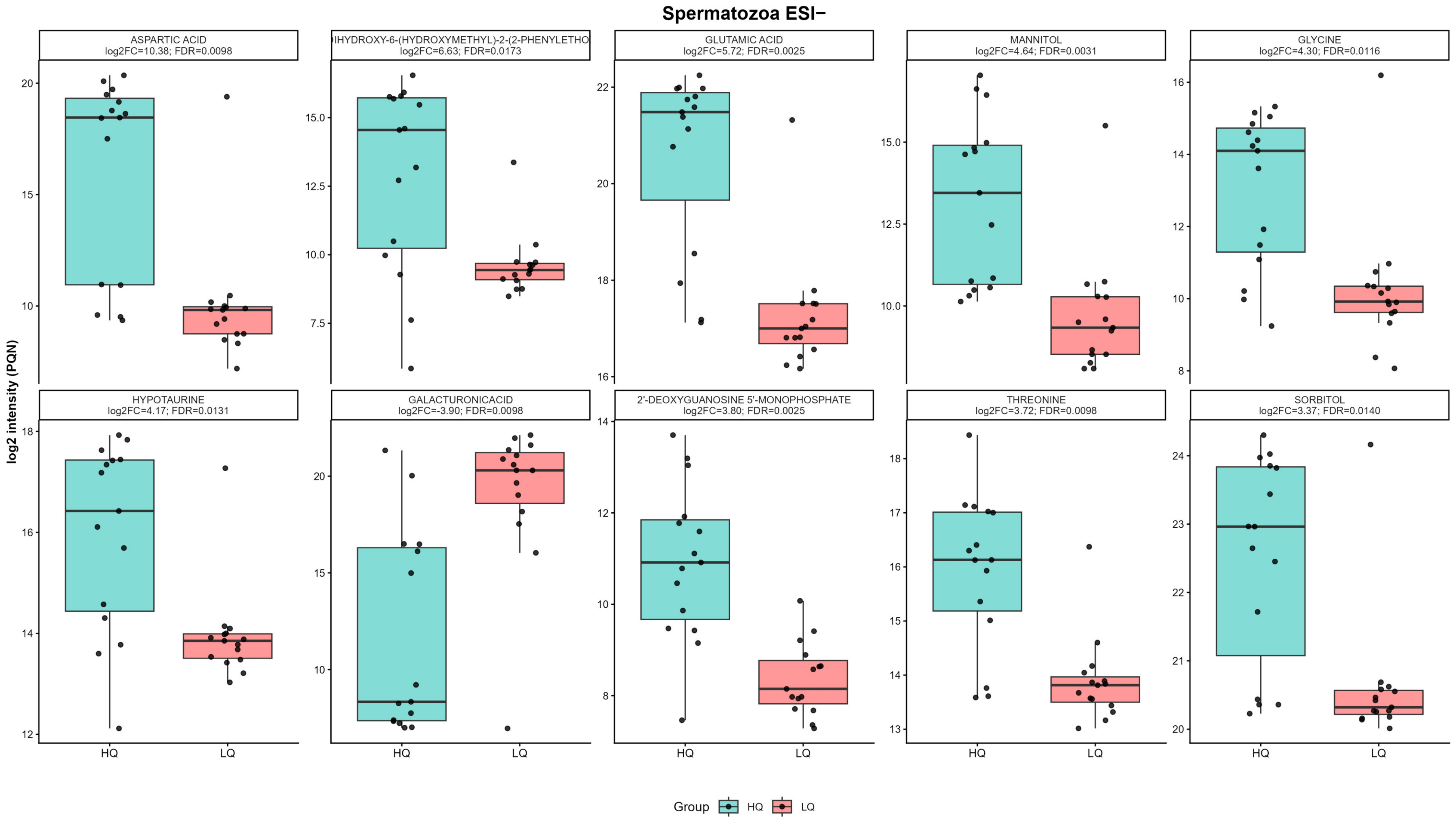

Spermatozoa ESI−
ASPARTIC ACID
log2FC=10.38; FDR=0.0098
DIHYDROXY-6-(HYDROXYMETHYL)-2-(2-PHENYLETHO
log2FC=6.63; FDR=0.0173
GLUTAMIC ACID
log2FC=5.72; FDR=0.0025
MANNITOL
log2FC=4.64; FDR=0.0031
GLYCINE
log2FC=4.30; FDR=0.0116
HYPOTAURINE
log2FC=4.17; FDR=0.0131
GALACTURONICACID
log2FC=-3.90; FDR=0.0098
2'-DEOXYGUANOSINE 5'-MONOPHOSPHATE
log2FC=3.80; FDR=0.0025
THREONINE
log2FC=3.72; FDR=0.0098
SORBITOL
log2FC=3.37; FDR=0.0140
log2 intensity (PQN)
HQ
LQ
Group
HQ
LQ

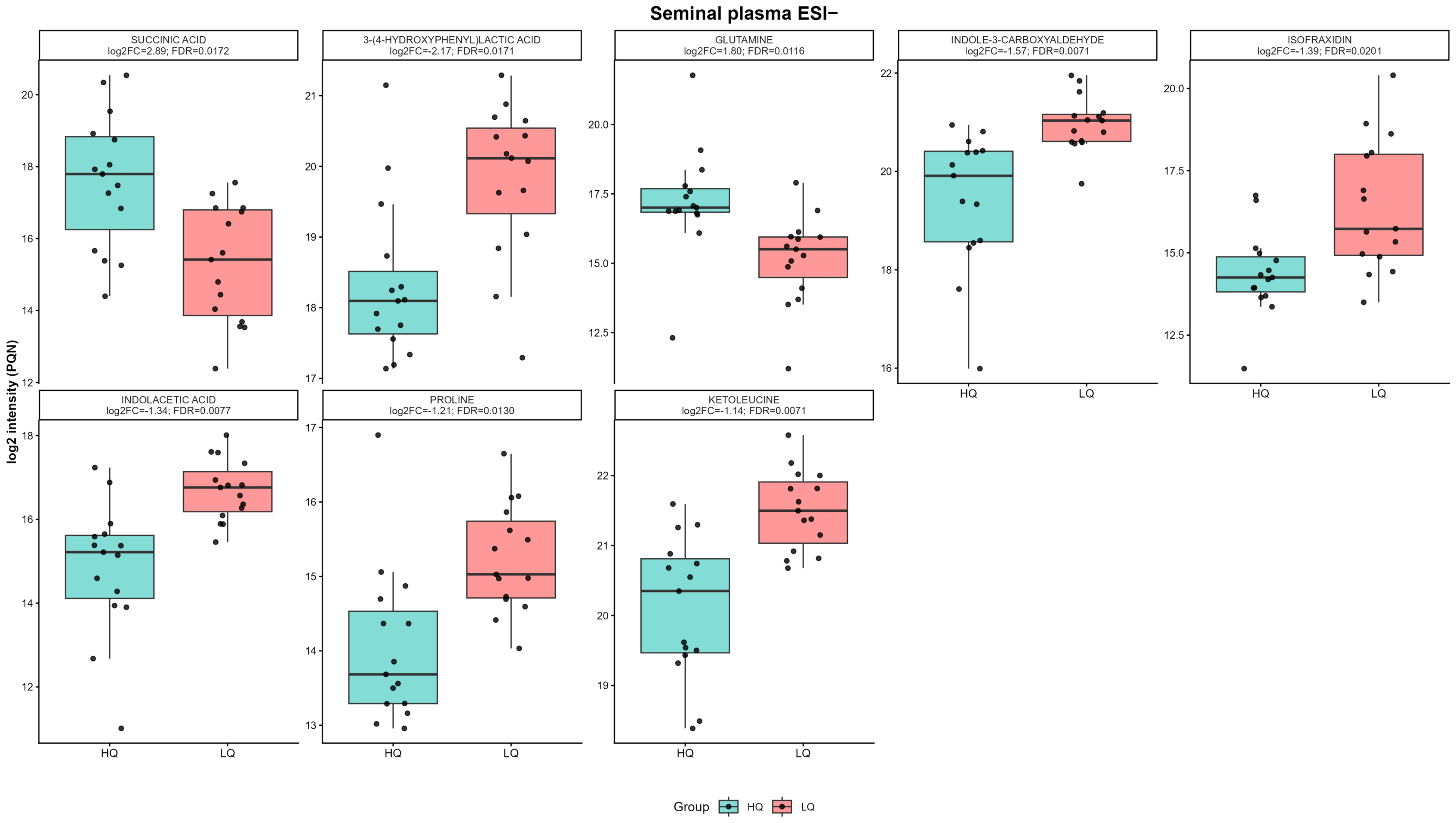
Seminal plasma ESI−
SUCCINIC ACID
log2FC=2.89; FDR=0.0172
3-(4-HYDROXYPHENYL)LACTIC ACID
log2FC=-2.17; FDR=0.0171
GLUTAMINE
log2FC=1.80; FDR=0.0116
INDOLE-3-CARBOXYALDEHYDE
log2FC=-1.57; FDR=0.0071
ISOFRAXIDIN
log2FC=-1.39; FDR=0.0201
INDOLACETIC ACID
log2FC=-1.34; FDR=0.0077
PROLINE
log2FC=-1.21; FDR=0.0130
KETOLEUCINE
log2FC=-1.14; FDR=0.0071
log2 intensity (PQN)
HQ
LQ
Group
HQ
LQ

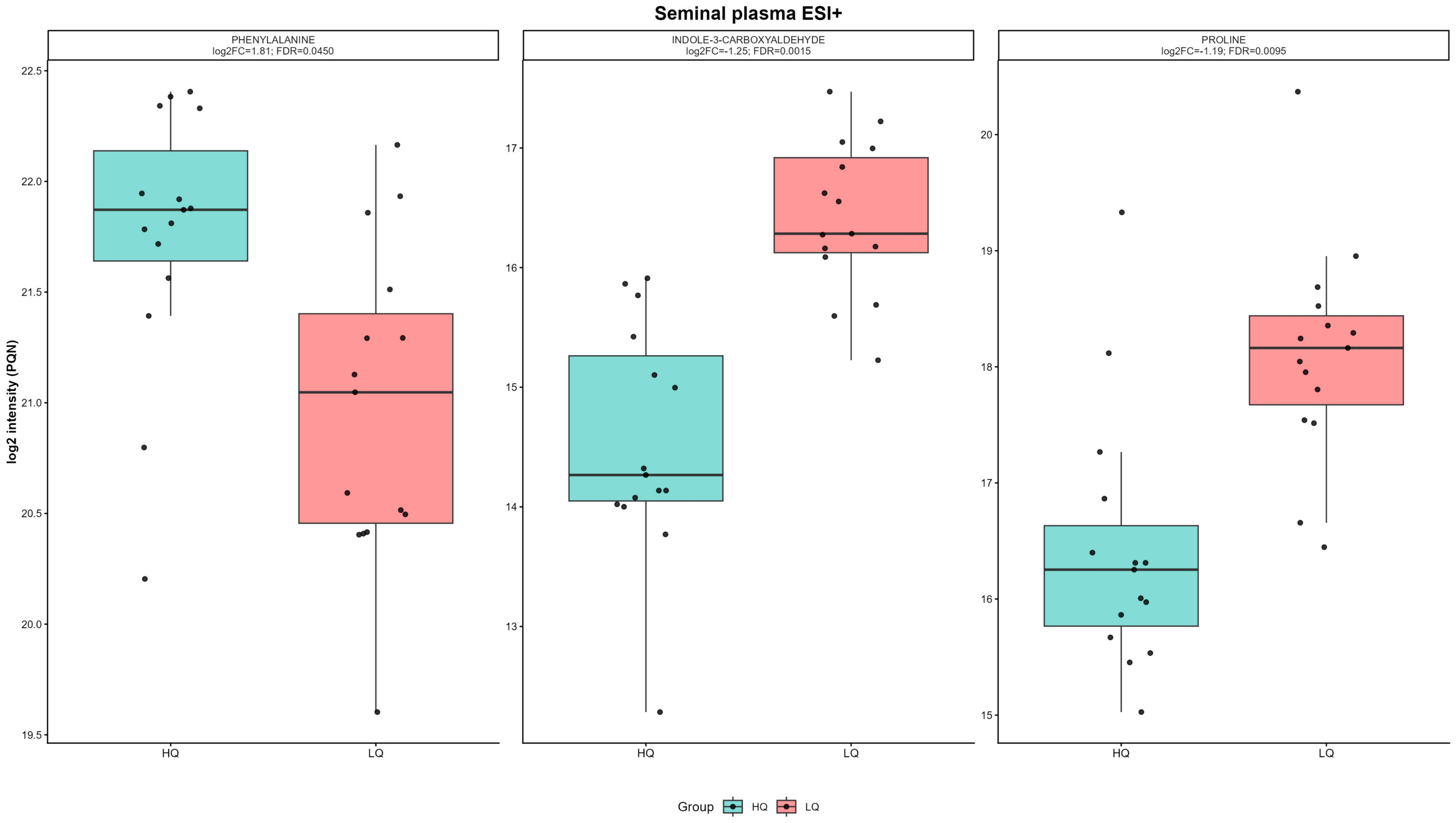
Seminal plasma ESI+
PHENYLALANINE
log2FC=1.81; FDR=0.0450
INDOLE-3-CARBOXYALDEHYDE
log2FC=-1.25; FDR=0.0015
PROLINE
log2FC=-1.19; FDR=0.0095
log2 intensity (PQN)
HQ
LQ
Group
HQ
LQ
