# Supplementary material for: Untargeted Metabolomic Profiling of Spermatozoa and Seminal Plasma Identifies Candidate Metabolic Indicators of Semen Quality in Holstein Bulls
Source: Animals (Basel). 2026 Oct 2;16(19):3098. doi: 10.3390/ani16193098 (PMC13663586; doi:10.3390/ani16193098)
Supplement: Supplementary file 1 [file animals-16-03098-s001.zip › Supplementary_Table_S1.pdf]

Supplementary Table S1.
Phenotypic characteristics and semen quality parameters of Holstein bulls included in the metabolomic study.

Parameter	HQ group (n = 15)	LQ group (n = 15)	p-value
Animal characteristics			
Age, years	3.1 ± 1.6	3.5 ± 1.6	0.52
Body weight, kg	856 ± 271	994 ± 290	0.19
Health status	Clinically healthy	Clinically healthy	-
Semen characteristics			
Number of ejaculates evaluated per bull	133.0 ± 52.0 (39-206)	113.9 ± 33.8 (44-169)	0.31
Ejaculate volume, mL	5.7 ± 1.2	6.7 ± 2.6	0.14
Sperm concentration, $\times 10^9$ cells/mL	1.66 ± 0.15	1.49 ± 0.25	0.04*
Activity score (0-8)	8.0 ± 0.1	5.8 ± 1.8	<0.001*
Rejection rate, %	4.4 ± 1.8	50.4 ± 20.1	<0.001*

Note: Data are presented as mean ± SD. P-values were calculated using the two-sided Mann–Whitney U test. All bulls were maintained under identical housing, feeding, and management conditions and received the same diet. Animal health was routinely monitored by veterinarians at the breeding center, and only clinically healthy bulls were included in the study. A single semen sample was collected from each bull over a one-month period during autumn for metabolomic analysis. Detailed records of progressive sperm motility, sperm morphology, abstinence interval, and field fertility outcomes were not available for the present metabolomic study. The activity score (0–8) routinely used at the breeding center includes assessment of sperm motility as one of its principal components.
