# Supplementary material for: Untargeted Metabolomic Profiling of Spermatozoa and Seminal Plasma Identifies Candidate Metabolic Indicators of Semen Quality in Holstein Bulls
Source: Animals (Basel). 2026 Oct 2;16(19):3098. doi: 10.3390/ani16193098 (PMC13663586; doi:10.3390/ani16193098)
Supplement: Supplementary file 1 [file animals-16-03098-s001.zip › Supplementary_Table_S3.pdf]

Supplementary Table S3.

The number of features at each filtering step is as follows:

Matrix	Initial features	After exogenous removal	After missing filter (>30%)	After duplicate aggregation	After prevalence filter (>50% in one group)	Final annotated metabolites
Spermatozoa ESI+	146	108	103	93	93	34
Spermatozoa ESI−	92	72	69	64	64	12
Seminal plasma ESI+	121	79	73	66	66	3
Seminal plasma ESI−	113	71	68	64	64	8

Note: All numbers reflect features detected after excluding blanks and QC samples. Duplicate entries were aggregated by selecting the variant with the lowest missing rate and highest mean intensity. NZV (near-zero variance) filtering was applied but did not remove additional metabolites; therefore, the column is not shown. PQN normalization and Pareto scaling were applied after these filtering steps. Final annotated metabolites = features meeting all selection criteria (FDR < 0.05, $|\log_2 FC| > 1$, VIP > 1, VIP stability > 70%, and AUC ≤ 0.20 or ≥ 0.80), i.e., the metabolites reported in Tables 1 and 3.
